# The down‐regulation of *MsWOX13‐2* promotes enhanced waterlogging resilience in alfalfa

**DOI:** 10.1111/tpj.70411

**Published:** 2025-08-20

**Authors:** Udaya Subedi, Kimberley Burton Hughes, Madeline Lehmann, Guanqun Chen, Surya Acharya, Abdelali Hannoufa, Cuong V. Nguyen, Stacy D. Singer

**Affiliations:** ^1^ Agriculture and Agri‐Food Canada, Lethbridge Research and Development Centre Lethbridge Alberta T1J 4B1 Canada; ^2^ Department of Agricultural, Food and Nutritional Science University of Alberta Edmonton Alberta T6G 2P5 Canada; ^3^ Agriculture and Agri‐Food Canada, London Research and Development Centre London Ontario N5V 4T3 Canada

**Keywords:** waterlogging tolerance, alfalfa, *MsWOX13‐2*, crop improvement, forage, *Medicago sativa*

## Abstract

Soil waterlogging events are predicted to escalate globally as a result of climate change, threatening the sustainability of alfalfa (*Medicago sativa* L.) and livestock production in the future. WUSCHEL‐related homeobox (WOX) transcription factors are known to play a role in numerous developmental processes and abiotic stress responses; however, their function in waterlogging resilience has not been investigated as of yet. In the present study, we functionally characterized the alfalfa *MsWOX13‐2* gene, which we found to be differentially expressed in response to waterlogging. Although the RNAi‐mediated silencing of *MsWOX13‐2* in alfalfa did not affect growth or morphology under normally watered conditions, *MsWOX13‐2* RNAi plants exhibited higher chlorophyll retention and maximum quantum efficiency of photosystem II, as well as greater survivability, compared to empty vector genotypes under waterlogging. Subsequent analyses indicated that *MsWOX13‐2* RNAi leaves accumulated less H_2_O_2_ and displayed a greater increase in superoxide dismutase activity under waterlogging, resulting in reduced oxidative damage, which may have contributed to the enhanced waterlogging tolerance in these genotypes. RNA‐Seq analysis confirmed alterations in the transcript levels of genes related to antioxidants, as well as those involved in photosynthesis, anaerobic fermentation, phytohormone‐related pathways, and transcriptional regulation in the leaves of *WOX13‐2* RNAi genotypes compared to wild type following waterlogging stress. Bi‐allelic mutation of *MsWOX13‐2* in alfalfa using CRISPR/Cas9 confirmed its function in waterlogging response. Overall, our findings suggest that MsWOX13‐2 acts as a negative regulator of waterlogging response in alfalfa, providing a novel candidate for downstream breeding endeavors in this important species.

## Significance Statement

While there is evidence that *WOX13* genes play a role in abiotic stress response in certain plant species, their function in the context of waterlogging stress has yet to be examined. In this study, we found that the alfalfa *MsWOX13‐2* gene acts as a negative regulator of waterlogging response in this species, providing important insight to facilitate downstream breeding endeavors.

## INTRODUCTION

Alfalfa (*Medicago sativa* L.) is a perennial forage legume of considerable importance in the livestock industry and is highly esteemed for its productivity, forage quality, nutrient content, and versatility in its use as hay, pasture, and silage. Its appeal is further bolstered by its many agronomic attributes, such as its deep‐rooted and perennial nature, as well as its ability to form symbiotic relationships with nitrogen‐fixing rhizobia (Singer et al., 2018). However, as with all crop species, it is often exposed to various environmental challenges in the field, which can hinder its production substantially. Alfalfa is particularly sensitive to soil waterlogging, which is predominantly caused by seasonal heavy rains, excessive irrigation, poor drainage, and an elevated water table (e.g., Barta & Sulc, 2002; Castonguay et al., 1993). Since the frequency and severity of waterlogging events are predicted to escalate globally as a result of climate change in coming years (Intergovernmental Panel on Climate Change (IPCC), 2021), this type of stress poses a significant threat to the sustainability of alfalfa and livestock production (Thivierge et al., 2023).

The excessive soil moisture levels encountered during waterlogging events result in a deprivation of molecular oxygen (O_2_) to plant cells (hypoxia), and in the case of roots, an eventual lack of O_2_ altogether (anoxia). These conditions lead to root damage and a consequent reduction in water and nutrient uptake (Ye et al., 2018), a decline in nodulation (Ploschuk et al., 2022), stomatal closure and a resultant decrease in photosynthesis (Anee et al., 2019), and a shift from aerobic respiration to anaerobic fermentation, which results in an ensuing accumulation of toxic compounds such as alcohols and aldehydes in the cytoplasm of plant cells (Ventura et al., 2020). In addition, as with most types of abiotic stress, waterlogging also triggers the increased production of reactive oxygen species (ROS; Hasanuzzaman et al., 2017), which serve as important signaling molecules during times of plant stress (Mittler et al., 2011). However, high levels of stress and/or prolonged exposure to stress typically lead to the production of excessive amounts of ROS, which cause cellular membrane damage via lipid peroxidation, as well as protein and nucleic acid impairment (Hasanuzzaman et al., 2020). These changes trigger various physiological and metabolic disturbances that inhibit growth and yield, and in severe cases, result in plant death (Naing & Kim, 2021).

To mitigate these impacts, at least to a certain degree, plants have evolved a series of sophisticated adaptive mechanisms. These include, for example, stress sensing and signaling strategies, as well as various morphological and physiological adjustments such as the increased production of aerenchyma and adventitious roots, alterations in phytohormone levels, and enhancements in the levels/activities of non‐enzymatic and enzymatic antioxidants, osmolytes, and enzymes with functions in anaerobic energy production (Barickman et al., 2019; Pan et al., 2022). Many of these alterations derive from changes in the expression of genes encoding specific transcription factors, including those belonging to the homeobox family (e.g., Cabello et al., 2016; Feyissa et al., 2021; Wei et al., 2019; Zeng et al., 2019).

Homeobox genes encode one of the largest superfamilies of transcription factors, including HD‐ZIP I through IV, KNOTTED‐1 like homeobox, BELL1‐like homeobox, and WUSCHEL‐related homeobox (WOX) families (Tsuda & Hake, 2016). The WOX family in particular comprises a group of plant‐specific transcription factors, all of which bear a homeodomain that is typically 60–66 amino acids long and can be classified into modern (present only in seed plants), intermediate (present only in vascular plants), and ancient (present in both vascular and non‐vascular plants) clades (van der Graaff et al., 2009). These transcription factors are involved in various processes in plants, including meristem maintenance (Ohmori et al., 2013; Wu et al., 2005), organ development (Deveaux et al., 2008; Lin et al., 2013), embryo patterning (Breuninger et al., 2008; Wu et al., 2007), callus formation (e.g., Ikeuchi et al., 2022), and abiotic stress response (e.g., Minh‐Thu et al., 2018; Zhu et al., 2004).

*WOX13* genes, which fall into the ancestral clade of the WOX family, have been found to be wound‐, phytohormone‐, and/or stress‐inducible; however, their particular expression patterns and responses to various phytohormones and types of stress appear to vary considerably among paralogs and species (e.g., He et al., 2019; Ikeuchi et al., 2022; Li et al., 2022; Minh‐Thu et al., 2018; Tanaka et al., 2023; Wang et al., 2019; Xu et al., 2023). In Arabidopsis, the single *AtWOX13* gene has been found to have myriad functions in tissue repair (Ikeuchi et al., 2022; Tanaka et al., 2023), the promotion of non‐meristematic cell fate (Ogura et al., 2023), replum formation (Romera‐Branchat et al., 2013), and floral transition (Deveaux et al., 2008). While there appears to be some functional overlap among *WOX13* genes in other plant species, especially with regard to flowering transition (e.g., Minh‐Thu et al., 2018), there seems to be a substantial amount of functional divergence among these genes (e.g., He et al., 2019; Zhang et al., 2022). In addition, although there is a distinct paucity of studies in this area, *WOX13* genes have also been implicated in drought tolerance in both rice (*Oryza sativa*; Minh‐Thu et al., 2018) and apple (*Malus xiaojinensis*; Lv et al., 2023), which highlights an important but understudied role for *WOX13* genes in abiotic stress response. In alfalfa, two *MsWOX13* genes were recently identified (*MsWOX13‐1* and *MsWOX13‐2*), and the expression of *MsWOX13‐1* was shown to be responsive to osmotic and cold stress (Xu et al., 2023). However, as of yet, nothing is known regarding the function of MsWOX13‐2 in alfalfa, and the role of *WOX13* genes in the context of waterlogging stress has yet to be evaluated.

As such, we endeavored to further our understanding of MsWOX13‐2 in alfalfa by examining its role in plant growth, morphology, and response to waterlogging stress. To achieve this, we assessed its transcriptional responsiveness to waterlogging treatment and generated alfalfa genotypes with reduced *MsWOX13‐2* expression levels via RNAi, as well as bi‐allelic mutation of *MsWOX13‐2* using CRISPR/Cas9‐mediated editing, which were then assessed for phenotypic variations, as well as waterlogging susceptibility. To further our understanding of the mechanisms at play in aboveground tissues that contribute to the enhancements in waterlogging tolerance observed in these genotypes, we also conducted a series of physiological, biochemical, and transcriptomic assessments of *MsWOX13‐2* RNAi genotypes. Our findings distinguish a new function for MsWOX13‐2 as a negative regulator of waterlogging response in alfalfa, providing a novel target for downstream breeding efforts aiming to improve waterlogging resilience in this crop.

## RESULTS

### *MsWOX13‐2* is down‐regulated under waterlogging stress

Four *MsWOX13‐2* alleles were identified in the tetraploid alfalfa cv. XinJiangDaYe genome (Chen et al., 2020) with chromosomal locations chr7.1;73,602,726‐73,603,463, chr7.2;75,652,796‐75,653,533, chr7.3;75,930,474‐75,931,211, and chr7.4;77,339,624‐77,340,361. These alleles corresponded to a region of the previously identified *MsWOX13‐2* gene in alfalfa cv. Zhongmu No. 1 (Xu et al., 2023) and comprised two exons and one intron, with a homeobox located within the second exon (corresponding to amino acids 64–131 in the predicted protein sequence; Figure S1a). The four alleles exhibited upwards of 97.6% identity between them at the nucleotide level within their coding regions and a minimum of 95.7% nucleotide identity with the coding region of the closely related *Medicago truncatula* homolog, Medtr7g023810.

**Figure 1:**
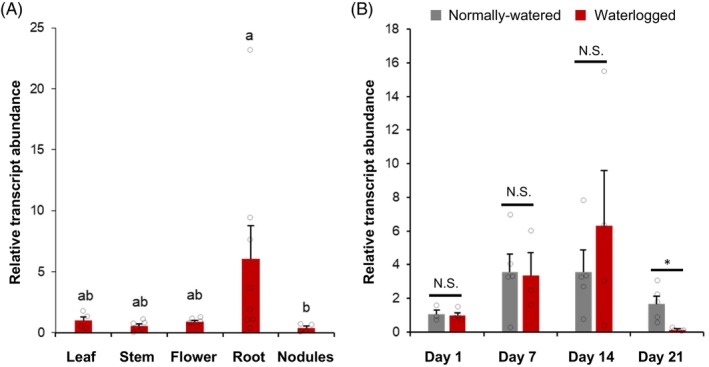
Gene expression patterns of *MsWOX13‐2*. (A) Tissue‐specific expression of *MsWOX13‐2* in alfalfa. (B) Transcriptional response of *MsWOX13‐2* to waterlogging stress in alfalfa. Blocks represent the mean of 4–8 biological replicates and bars indicate standard errors. Lower case letters indicate statistically significant differences between groups within a treatment as determined by the Kruskal–Wallis test followed by Dunn's multiple comparison test with ‘Bonferroni’ adjustment (*P* < 0.05). * and N.S. denote significant differences at *P* ≤ 0.05 or no significant difference, respectively, between normally watered and waterlogged conditions as determined by two‐tailed *t*‐tests (assuming unequal variance).

To gain further insight into the tissue‐specific expression patterns of *MsWOX13‐2* in the wild‐type N4.4.2 alfalfa genotype used in this study, qRT‐PCR assays were performed using RNA derived from leaves, stems, flowers, roots, and nodules. While *MsWOX13‐2* transcripts tended to preferentially accumulate in roots (excluding nodules) compared to the other tissues tested in this study, only nodules exhibited significantly lower transcript levels than roots (Figure 1A). Similarly, qRT‐PCR assays were also performed using RNA derived from leaves of the wild‐type genotype grown under normally watered and waterlogged conditions to evaluate the waterlogging responsiveness of this gene. Although *MsWOX13‐2* expression did not differ significantly among plants grown under normally watered and waterlogged conditions after 1, 7, and 14 days of treatment, a significant, approximately 13‐fold reduction in *MsWOX13‐2* transcript levels was observed after 21 days of waterlogging compared to plants grown under normally watered conditions (Figure 1B).

### *MsWOX13‐2* RNAi genotypes exhibit the specific down‐regulation of *MsWOX13‐2*

*MsWOX13‐2* RNAi and empty vector alfalfa genotypes were generated (Figure S1b,c), and qRT‐PCR was used to confirm the down‐regulation of *MsWOX13‐2* in RNAi genotypes. Genotypes RNAi‐203 and RNAi‐205 (derived from independent transformation events) exhibited the greatest levels of down‐regulation, with approximately 17‐fold and 4‐fold reductions in *MsWOX13‐2* transcript levels compared to wild‐type, respectively (Figure S1d), and were thus used for all subsequent experiments. Despite the fact that within the target region of our RNAi vector, *MsWOX13‐2* only exhibited 62.5% nucleotide identity with *MsWOX13‐1* and would thus not be expected to be inadvertently down‐regulated in our RNAi genotypes, we also conducted qRT‐PCR analysis using total RNA from RNAi‐203 and RNAi‐205 genotypes as template, as well as the wild‐type control, to ensure that the expression of *MsWOX13‐1* was not impacted. As expected, no significant differences in *MsWOX13‐1* transcript levels were evident among genotypes (Figure S1e), confirming the specificity of our RNAi vector.

### Silencing of *MsWOX13‐2* does not impact nodulation

Previously, *MsWOX13‐2* was found to be expressed at high levels in the nodules of alfalfa cv. ZhongmuNo.1 (Xu et al., 2023), which contrasted with results in the current study with the N4.4.2 alfalfa genotype (Figure 1A). To further evaluate the role of MsWOX13‐2 in the context of nodules, assessments were carried out to determine whether the suppression of *MsWOX13‐2* affected nodulation in alfalfa following inoculation with rhizobia. No alterations in total nodule numbers, nodule size, or color (Figure S2a,b) were observed among *MsWOX13‐2* RNAi and wild‐type genotypes under normally watered conditions in this study. In line with this, no differences were observed in terms of chlorophyll content or plant height among genotypes when grown under nitrogen‐deprived or nitrogen‐abundant conditions (Figure S2c–e). Similarly, while a paucity of nodule primordia formed under nitrogen‐supplied conditions across genotypes, all genotypes developed numerous phenotypically normal nodules under nitrogen‐deprived conditions (Figure S2f), suggesting that MsWOX13‐2 does not play an important role in nodulation in the N4.4.2 genotype.

### The down‐regulation of *MsWOX13‐2* in alfalfa promotes waterlogging resilience

**Figure 2:**
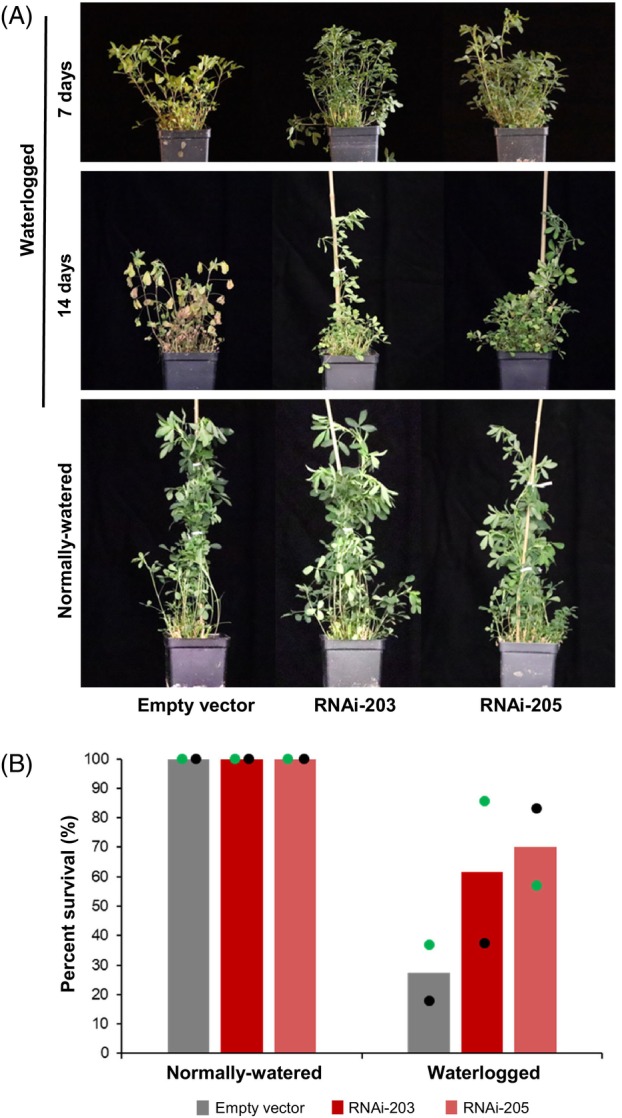
The RNAi‐mediated silencing of *MsWOX13‐2* leads to improved waterlogging tolerance in alfalfa. (A) Representative images of plants grown under normally watered conditions, as well as those subjected to waterlogging for 7 and 14 days. (B) Survival rate of empty vector and *MsWOX13‐2* RNAi genotypes under normally watered and waterlogged conditions. Blocks represent mean values of survival rates for two experimental sets. Green dots represent data points from set 1 and black dots represent data points from set 2. In each experimental set, 18 biological replicates of three independent empty vector genotypes (pooled), and 5–7 biological replicates of RNAi‐203 and RNAi‐205 genotypes, respectively, were utilized for assessment.

To determine whether the down‐regulation of *MsWOX13‐2* had any impact on susceptibility to waterlogging stress, we evaluated the stress symptoms and growth of *MsWOX13‐2* RNAi and empty vector genotypes subjected to normally watered and waterlogged conditions. Under waterlogged conditions, leaf chlorosis became apparent after approximately 1 week in empty vector genotypes, while leaf wilting and senescence were observed following approximately 2 weeks of waterlogging stress (Figure 2A). Interestingly, these symptoms were far less pronounced in both *MsWOX13‐2* RNAi genotypes than in empty vector genotypes, which hints at the possibility that these plants were better able to withstand this type of stress. In order to evaluate whether the *MsWOX13‐2* RNAi genotypes exhibited superior survival after waterlogging stress followed by subsequent re‐oxygenation, RNAi and empty vector genotypes were subjected to 14 days of waterlogging, after which time the water was drained and plants were maintained under a normal watering regime for 6 weeks. While only 27% of empty vector plants survived this treatment, 62 and 70% of RNAi‐203 and RNAi‐205 genotypes survived, respectively (Figure 2B).

**Figure 3:**
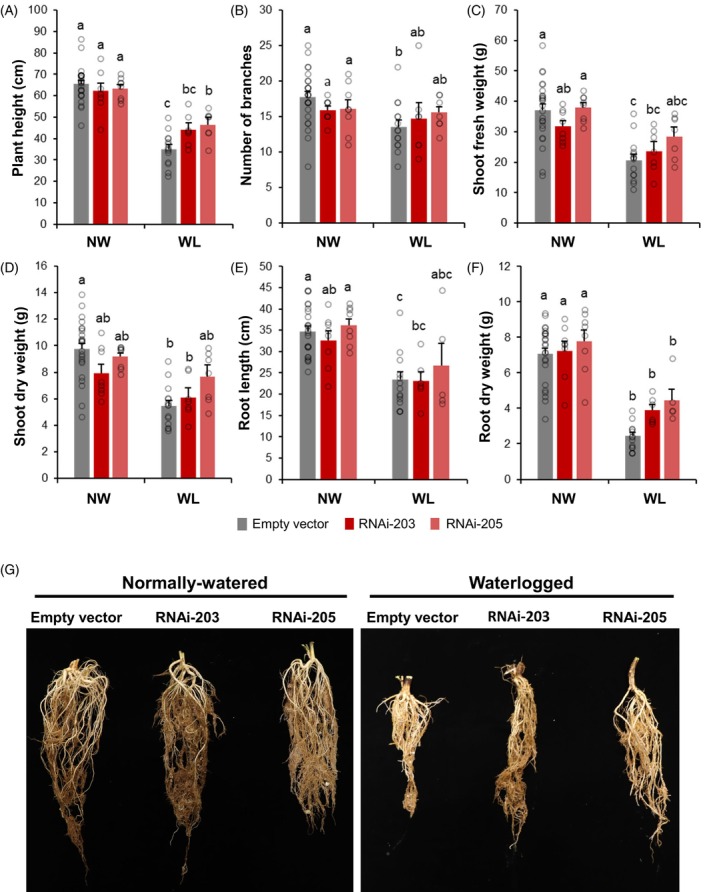
Morphological evaluation of empty vector and *MsWOX13‐2* RNAi genotypes under normally watered (NW) and waterlogged (WL) conditions. (A) Plant height, (B) number of branches, (C) shoot fresh weight, (D) shoot dry weight, (E) root length, and (F) root dry weight of empty vector and *MsWOX13‐2* RNAi genotypes measured under both NW and WL conditions. (G) Representative images of empty vector and *MsWOX13‐2* RNAi roots under NW conditions and after 14 days of waterlogging. Blocks represent mean values of 18–24 biological replicates of three independent empty vector genotypes (pooled), and 7–8 biological replicates of each *MsWOX13‐2* RNAi genotype, respectively. Bars indicate standard errors. Lower case letters denote statistically significant differences between groups as determined by two‐way anova followed by Tukey's HSD test (*P* ≤ 0.05).

In terms of plant growth, under normally watered conditions, no obvious differences were observed among *MsWOX13‐2* RNAi and empty vector genotypes in any of the shoot or root parameters assessed (Figures 2A and 3). Following 2 weeks of waterlogging, however, empty vector genotypes exhibited significant reductions in plant height, the number of primary branches, and shoot fresh and dry weight relative to their normally watered counterparts (Figure 3A–D). Conversely, in the case of both *MsWOX13‐2* RNAi genotypes evaluated, while declines in these parameters were also noted under waterlogging, these differences were not significant with the exception of plant height (Figure 3A–D).

In terms of root characteristics, significant differences in root length were not observed among genotypes under either growth condition. However, a significant reduction in root length was observed in empty vector genotypes, but not *MsWOX13‐2* RNAi genotypes, under waterlogged compared to normally watered conditions (Figure 3E). In contrast, a notable and significant decline was observed in root dry weight across *MsWOX13‐2* RNAi and empty vector genotypes under waterlogged compared to normally watered conditions (Figure 3F,G). Moreover, no obvious aerenchyma were observed in any genotype, even after 28 days of waterlogging, suggesting that aerenchyma may not play important roles in the waterlogging tolerance observed in *MsWOX13‐2* RNAi genotypes (Figure S3).

### Silencing of *MsWOX13‐2* leads to the maintenance of higher chlorophyll contents and *F*_v_/*F*_m_ under waterlogged conditions

**Figure 4:**
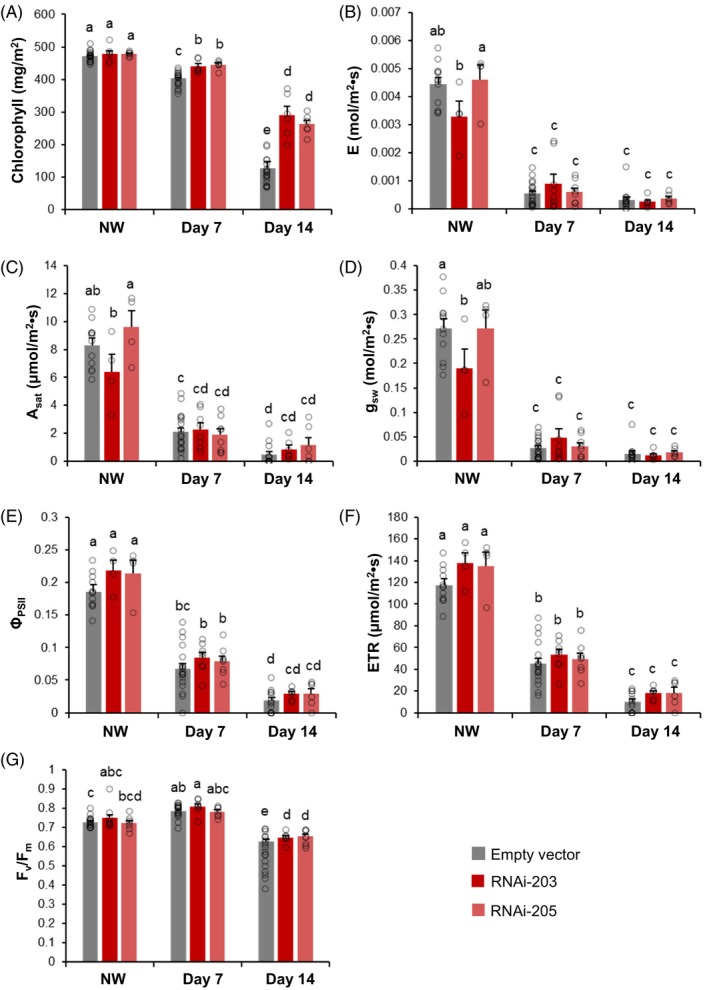
Photosynthesis‐related characteristics in empty vector and *MsWOX13‐2* RNAi genotypes under normally watered (NW) and waterlogged conditions. (A) Chlorophyll content, (B) transpiration rate (*E*), (C) light‐saturated photosynthetic rate (*A*_sat_), (D) stomatal conductance to water vapor (*g*_sw_), (E) quantum yield of photosystem II (*Φ*_PSII_), (F) electron transfer rate at photosystem II (PSII), and (G) maximum quantum efficiency of PSII (*F*_v_/*F*_m_) in empty vector and *MsWOX13‐2* RNAi genotypes under NW conditions and after waterlogging for 7 and 14 days. Blocks represent mean values of 12–18 biological replicates of three independent empty vector genotypes (pooled), and 4–8 biological replicates of each *MsWOX13‐2* RNAi genotype, respectively. Bars indicate standard errors. Lower case letters denote statistically significant differences between groups as determined by two‐way anova followed by Tukey's HSD test (*P* ≤ 0.05).

*MsWOX13‐2* RNAi and empty vector genotypes displayed no significant differences in leaf chlorophyll content under normally watered conditions. However, chlorophyll content decreased progressively with the length of waterlogging stress in all genotypes, with the decline being less substantial in both *MsWOX13‐2* RNAi genotypes compared to empty vector genotypes (Figure 4A). Indeed, following 14 days of waterlogging, both RNAi genotypes exhibited chlorophyll contents that were more than double those of empty vector genotypes (Figure 4A).

In terms of other photosynthesis‐related parameters, transpiration rate (*E*), stomatal conductance to water vapor (*g*_sw_), electron transport rate (ETR), and the quantum yield of photosystem II (*Φ*_PSII_) did not differ significantly among *MsWOX13‐2* RNAi and empty vector genotypes under normally watered or waterlogged conditions (Figure 4B,D–F). Light‐saturated photosynthetic rate (*A*_sat_), on the contrary, decreased significantly between days 7 and 14 of waterlogging in empty vector genotypes, but not in either of the *MsWOX13‐2* RNAi genotypes (Figure 4C). In contrast, although maximum quantum efficiency of photosystem II (*F*_v_/*F*_m_) decreased significantly in all genotypes at day 14 compared to day 7 of waterlogging treatment, this decrease was less severe in the RNAi genotypes, and they exhibited significantly higher *F*_v_/*F*_m_ values than empty vector genotypes following 14 days of waterlogging (Figure 4G).

### Silencing of *MsWOX13‐2* attenuates H_2_O_2_ accumulation and oxidative damage under waterlogged conditions

**Figure 5:**
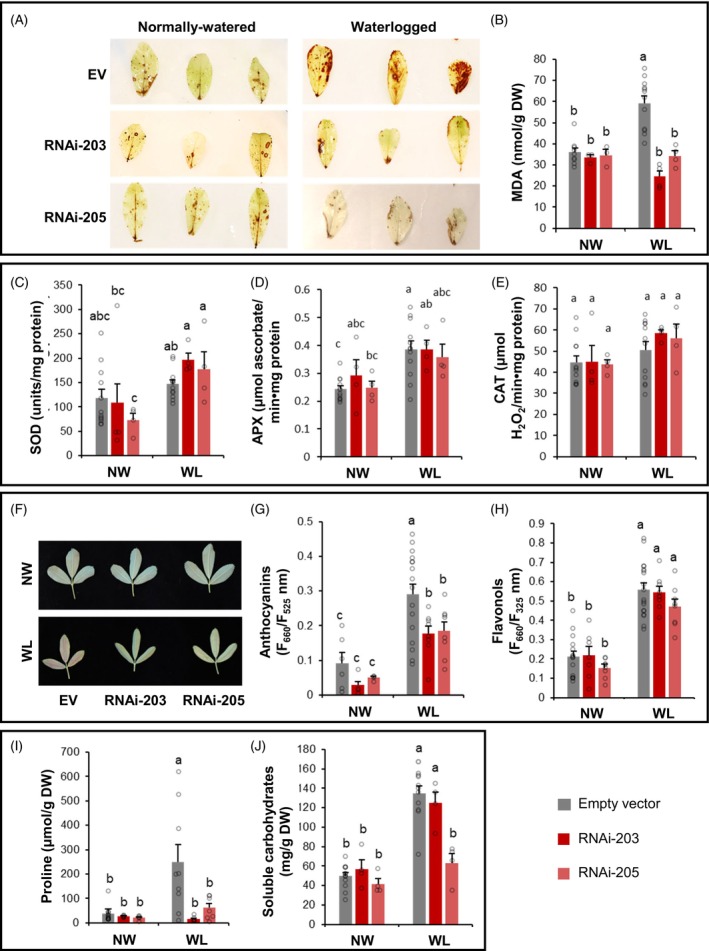
Antioxidant and osmolyte characteristics in the leaves of empty vector and *MsWOX13‐2* RNAi genotypes under normally watered (NW) and waterlogged (WL) conditions. (A) Representative images of leaflets from empty vector and *MsWOX13‐2* RNAi genotypes following DAB staining. (B) Malondialdehyde levels, (C) superoxide dismutase activity, (D) ascorbate peroxidase activity, (E) and catalase activity in the leaves of alfalfa genotypes under WL and NW conditions. (F) Representative images of trifoliate leaves from empty vector and *MsWOX13‐2* RNAi genotypes 14 days after the initiation of waterlogging, (G) anthocyanin index, (H) flavonol index, (I) proline levels, and (J) soluble carbohydrate levels in the leaves of alfalfa genotypes grown under NW and WL conditions. Blocks represent the mean values of 9–18 biological replicates of three independent empty vector genotypes (pooled), and 3–8 biological replicates of each *MsWOX13‐2* RNAi genotype, respectively. Bars indicate standard errors. Lower case letters denote statistically significant differences between groups as determined by two‐way anova followed by Tukey's HSD test (*P* ≤ 0.05). EV, empty vector genotypes.

To examine the extent of ROS accumulation in *MsWOX13‐2* RNAi and empty vector genotypes, leaves were stained with 3,3′ diaminobenzene (DAB) as a means of visualizing H_2_O_2_. While no apparent differences in DAB staining intensity were observed among genotypes under normally watered growth conditions, increased staining intensity was noted in empty vector genotypes, but not *MsWOX13‐2* RNAi genotypes, under waterlogging stress (Figure 5A).

To determine whether increased H_2_O_2_ accumulation in empty vector genotypes under waterlogged conditions corresponded with elevated levels of oxidative damage, malondialdehyde (MDA) levels were assessed in leaves from plants grown under both normally watered and waterlogged conditions. While no significant differences in MDA levels were observed among genotypes under normal growth conditions, empty vector genotypes, but not *MsWOX13‐2* RNAi genotypes, exhibited a significant 64% relative increase in MDA levels under waterlogging compared to normally watered conditions (Figure 5B). In line with this, MDA levels were significantly lower in *MsWOX13‐2* RNAi genotypes than in empty vector genotypes following waterlogging (Figure 5B).

### Silencing of *MsWOX13‐2* causes differential accumulation and activities of antioxidants under waterlogged conditions

To determine whether the difference in oxidative damage between *MsWOX13‐2* RNAi and empty vector genotypes under waterlogging might be attributed, at least in part, to modulations in antioxidants, we first assessed the enzymatic activities of superoxide dismutase (SOD), ascorbate peroxidase (APX), and catalase (CAT) in leaves from plants grown under normally watered and waterlogged conditions. No significant differences in the activities of any of these three enzymes were observed among genotypes grown under normal growth conditions (Figure 5C–E). However, when exposed to waterlogging stress, SOD activity in both *MsWOX13‐2* RNAi genotypes, but not empty vector genotypes, increased significantly compared to normally watered conditions (80.1 and 141.5% relative increase in RNAi‐203 and RNAi‐205, respectively; Figure 5C). In contrast, APX activity increased significantly in empty vector genotypes, but not *MsWOX13‐2* RNAi genotypes, under waterlogging compared to normally watered conditions (Figure 5D). No differences were noted in CAT activity across growth conditions in any genotype (Figure 5E).

Levels of anthocyanins and flavonols, which are non‐enzymatic antioxidants, were also examined in the leaves of *MsWOX13‐2* RNAi and empty vector genotypes under both normally watered and waterlogged growth conditions. While the leaves of empty vector genotypes developed a distinct red coloration, which is characteristic of anthocyanin accumulation, following 14 days of waterlogging, this characteristic was far less pronounced in the RNAi genotypes (Figure 5F). This correlated well with results from the quantitative measurement of anthocyanin levels in leaves, which indicated that although anthocyanins increased significantly in all genotypes under waterlogging treatment compared to normally watered conditions, levels were significantly lower in both *MsWOX13‐2* RNAi genotypes compared to empty vector genotypes under waterlogging (41.4 and 36.5% relative decrease in RNAi‐203 and RNAi‐205, respectively; Figure 5G). Conversely, a significant increase in flavonols was observed under waterlogged compared to normally watered growth conditions in all genotypes, and no significant differences were observed among genotypes under either growth condition (Figure 5H).

### Silencing of *MsWOX13‐2* diminishes osmolyte accumulation under waterlogging stress

The levels of proline and soluble carbohydrates were also evaluated in leaf tissues from plants grown under both waterlogged and normally watered conditions to examine the possible roles of osmolytes in the improved waterlogging tolerance observed in *MsWOX13‐2* RNAi genotypes. Neither proline nor soluble carbohydrate levels differed significantly among genotypes under normally watered growth conditions (Figure 5I,J). However, while the levels of both proline and soluble carbohydrates increased significantly in empty vector genotypes under waterlogging stress, this was not consistently the case in *MsWOX13‐2* RNAi genotypes (Figure 5I,J).

### Comparative RNA‐Seq analysis of wild‐type and *MsWOX13‐2* RNAi genotypes grown under normally watered and waterlogged conditions

Due to the fact that we observed waterlogging responsiveness of *MsWOX13‐2* in alfalfa leaves, as well as significant distinctions in physiological and biochemical characteristics in the leaves of *MsWOX13‐2* RNAi genotypes under waterlogging, we sought to further our understanding of the transcriptional changes occurring in the leaves of *MsWOX13‐2* RNAi genotypes. To achieve this, we conducted comparative RNA‐Seq analysis using total RNA extracted from the leaves of wild‐type alfalfa, as well as RNAi‐203 and RNAi‐205 genotypes, grown under normally watered and waterlogged conditions. Samples yielded an average of 128 380 311 raw reads and 125 912 169 clean reads (Table S1). Principal component analysis (PCA) using fragments per kilobase of transcript per million mapped reads (FPKM) values revealed a distinct clustering of samples into three groups, including a group comprising all normally watered plants (RNAi‐203, RNAi‐205, and wild‐type), a second consisting of the RNAi‐203 and RNAi‐205 genotypes under waterlogging, and a third comprising the wild‐type genotype under waterlogging (Figure S4a), confirming the similarity of biological replicates between treatment groups.

Since PCA analysis indicated that the RNAi‐203 and RNAi‐205 genotypes clustered tightly together, we assessed these two genotypes collectively for all remaining RNA‐Seq analyses. In the case of wild‐type and *MsWOX13‐2* RNAi genotypes, a total of 11 500 (5671 up‐regulated and 5829 down‐regulated) and 12 718 (6343 up‐regulated and 6375 down‐regulated) differentially expressed genes (DEGs), respectively, were identified between normally watered and waterlogged conditions (Figure S4b; Data S1). Similarly, under normally watered and waterlogged conditions, a total of 2262 (1203 up‐regulated and 1059 down‐regulated) and 12 122 (5850 up‐regulated and 6272 down‐regulated) DEGs were identified, respectively, between wild‐type and *MsWOX13‐2* RNAi genotypes (Figure S4b; Data S1).

Cluster analysis of the DEGs identified in this study also revealed tight clustering among biological replicate samples and distinct clustering of wild‐type and *MsWOX13‐2* RNAi genotypes under waterlogged conditions, as well as between normally watered and waterlogged conditions for all genotypes (Figure S4c). Taken together, these findings suggest that transcriptional variations between wild‐type and *MsWOX13‐2* RNAi alfalfa genotypes are far more prevalent under waterlogging stress than under normally watered growth conditions. Furthermore, qRT‐PCR validation of 10 stress‐related DEGs indicated a strong consensus with our RNA‐Seq results, providing a correlation coefficient value of 0.965, thus supporting the accuracy of the RNA‐Seq data (Figure S4d).

### GO term enrichment analysis of DEGs

To investigate the possible roles of DEGs identified through RNA‐Seq, gene ontology (GO) term enrichment analyses were conducted between wild‐type and *MsWOX13‐2* RNAi genotypes under normally watered and waterlogged conditions. Under normally watered conditions, DEGs identified between wild‐type and *MsWOX13‐2* RNAi genotypes were significantly enriched in 10 metabolic function category terms, including several related to hydrolase, oxidoreductase, and peptidase activity (Figure S5a). In contrast, no GO terms were significantly enriched in the cellular component category, and the only GO term significantly enriched in the biological process category was ‘defense response’ (Figure S5a).

Under waterlogged conditions, five significantly enriched terms were identified between wild‐type and *MsWOX13‐2* RNAi genotypes in the metabolic function category, including ‘calcium ion binding’ and ‘signal transducer activity’, as well as several related to hydrolase, oxidoreductase, and peptidase activity (Figure S5b). Conversely, 14 GO terms within the cellular component category were significantly enriched between genotypes, including various terms related to photosynthesis, as well as ‘oxidoreductase complex’, for example (Figure S5b). Interestingly, no terms were significantly enriched in the biological process category between genotypes under waterlogged conditions (Figure S5b).

### Differential transcriptional responses in general metabolic pathways between wild‐type and *MsWOX13‐2* RNAi genotypes

**Figure 6:**
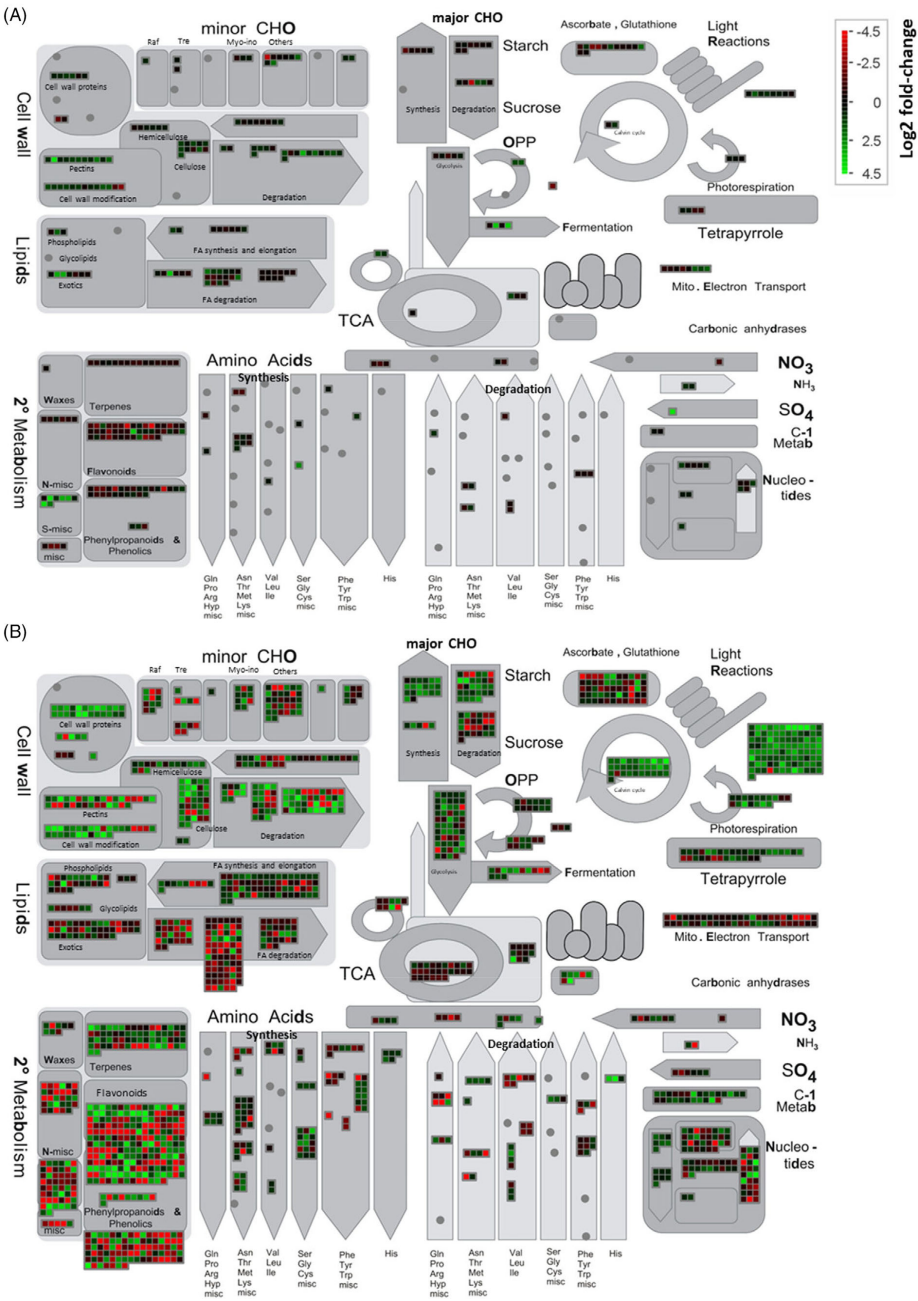
Transcriptional changes in various metabolic pathways in *MsWOX13‐2* RNAi genotypes compared to wild‐type. (A) Transcriptional changes under normally watered conditions, and (B) transcriptional changes after waterlogging for 14 days. Boxes depict the extent of log_2_ fold changes in significantly up‐regulated (green) or down‐regulated (red) differentially expressed genes in each pathway. Myo‐ino, myo‐inositol metabolism; Raf, raffinose metabolism; Tre, trehalose metabolism.

To gain a deeper insight into the differential transcriptional responses between wild‐type and *MsWOX13‐2* RNAi genotypes, Mapman pathway analysis was performed using DEGs identified between wild‐type and *MsWOX13‐2* RNAi genotypes under normally watered and waterlogged conditions. As was the case for the overall number of DEGs identified between genotypes, a higher number of DEGs involved in general metabolic pathways were found under waterlogged than normally watered conditions (Figure 6; Data S2). As such, subsequent pathway analyses were directed toward the investigation of differences between wild‐type and *MsWOX13‐2* RNAi genotypes under waterlogged conditions.

#### Abiotic stress‐ and photosynthesis‐related pathways

Interestingly, under waterlogging, a slightly higher number of DEGs (65 of 106) related to abiotic stress response were down‐regulated in *MsWOX13‐2* RNAi genotypes compared to wild‐type (Figure S6a; Data S2). Conversely, the vast majority of DEGs within photosynthesis‐related pathways were significantly up‐regulated in *MsWOX13‐2* RNAi genotypes compared to wild‐type (Figure 6B; Data S2). For example, all but one DEG within the light reactions bin, including 10 DEGs encoding chlorophyll a‐b binding proteins, were up‐regulated in *MsWOX13‐2* RNAi genotypes compared to wild‐type (Figure 6B; Data S2). Similarly, all but one DEG falling within the Calvin cycle bin were up‐regulated in *MsWOX13‐2* RNAi genotypes compared to wild‐type, including Medtr7g007230, Medtr7g007210, and Medtr4g127870, which encode the small subunit of ribulose 1,5‐bisphosphate carboxylase (Figure 6B; Data S2).

#### Pathways related to energy production

Substantial alterations in the transcription of genes related to energy production were also noted between RNAi and wild‐type genotypes under waterlogging. For example, 40 of 54 glycolysis‐related DEGs were expressed at elevated levels in *MsWOX13‐2* RNAi genotypes compared to wild‐type (Figure 6B; Data S2). These genes primarily encoded enzymes involved in carbohydrate substrate exchange, including fructose‐2,6‐bisphosphatase (Medtr5g071900), aldolase (Medtr5g096670), phosphoglycerate kinase (Medtr2g066110), pyruvate kinase (Medtr4g083340), phosphoenolpyruvate carboxylase (Medtr1g094000), phosphoglucomutase (Medtr1g102035), and phosphoglycerate mutase (Medtr4g055260 and Medtr8g091330; Data S2). In addition, 8 of 13 DEGs with functions in fermentation were also expressed at higher levels in *MsWOX13‐2* RNAi genotypes compared to wild‐type under waterlogging (Figure 6B; Data S2). For example, a gene encoding lactate dehydrogenase (LDH; Medtr4g101130), as well as three genes encoding alcohol dehydrogenase (ADH; Medtr3g089940, Medtr3g089970, and Medtr3g089977), were significantly up‐regulated in *MsWOX13‐2* RNAi genotypes compared to wild‐type under waterlogged conditions (Data S2). Conversely, a single gene encoding pyruvate decarboxylase (PDC; Medtr6g004200) was found to be expressed at reduced levels in the RNAi genotypes under waterlogging stress (Data S2).

#### Redox‐related pathways

Under waterlogging stress, genes with functions in heme, thioredoxin, ascorbate/glutathione, and glutaredoxin metabolism were fairly evenly distributed in terms of up‐ and down‐regulation in *MsWOX13‐2* RNAi genotypes compared to wild‐type. In contrast, all DEGs falling within peroxiredoxin and dismutase/CAT bins exhibited significantly higher levels of expression in RNAi genotypes compared to wild‐type (Figure S6b; Data S2). Intriguingly, DEGs encoding various SOD enzymes, including Medtr1g048990 (SOD [Fe]), Medtr3g094250 (SOD [Mn]), and Medtr4g057240 (SOD [Cu‐Zn]), displayed significantly elevated expression levels in RNAi genotypes compared to wild‐type under waterlogging (Data S2).

In terms of non‐enzymatic antioxidants, DEGs associated with flavonoid metabolism were up‐ and down‐regulated in *MsWOX13‐2* RNAi genotypes compared to wild‐type under waterlogging conditions to a fairly equal extent (Figure 6B; Data S2). Notably, 17 of 18 DEGs encoding chalcone synthases (CHS; e.g., Medtr1g098140), which is the first committed enzyme in flavonoid biosynthesis, were substantially down‐regulated in *MsWOX13‐2* RNAi genotypes compared to wild‐type under waterlogged conditions. Furthermore, approximately two thirds of DEGs (46 of 66) involved in terpene metabolism, which in general includes genes related to tocopherol biosynthesis (e.g., Medtr1g090913 encoding phytol kinase), carotenoid biosynthesis (e.g., Medtr3g450510 and Medtr2g040060 encoding phytoene synthase and LUTEIN‐DEFICIENT 2, respectively), and terpenoid biosynthesis (e.g., Medtr3g052120 and Medtr4g005190 encoding terpene synthase), were expressed at higher levels in RNAi genotypes compared to wild‐type under waterlogging (Figure 6B; Data S2).

#### Transcription factor‐encoding DEGs

A large number of DEGs (1046) identified between *MsWOX13‐2* RNAi genotypes and wild‐type under waterlogging stress encoded transcription factors (Figure S6c; Data S2). Among these, the most prominent were WRKY, bHLH, MYB, AP2‐EREBP, HB, bZIP, C_2_H_2_, GRAS, NAC, Aux/IAA, ARF, and PHOR1 family members. While the majority of DEGs within bHLH, AP2‐EREBP, HB, Aux/IAA, and ARF families exhibited significantly higher expression levels under waterlogging stress in *MsWOX13‐2* RNAi genotypes than wild‐type, most DEGs within WRKY, bZIP, NAC, and PHOR1 families displayed lower levels of expression in RNAi genotypes (Figure S6c; Data S2). Conversely, the number of DEGs within MYB, C_2_H_2_, and GRAS families were up‐ and down‐regulated to a relatively equivalent extent (Figure S6c; Data S2).

#### Phytohormone‐related pathways

In the case of phytohormones, the majority of DEGs (41 of 69) associated with ethylene‐related pathways were down‐regulated in *MsWOX13‐2* RNAi genotypes compared to wild‐type under waterlogging stress (Figure S6d; Data S2). For example, all four DEGs encoding 1‐aminocyclopropane‐1‐carboxylic acid synthase (ACC synthase; Medtr6g091760, Medtr7g079080, Medtr8g098930 and Medtr8g028600), which catalyzes the conversion of S‐adenosyl‐L‐methionine (SAM) into ACC, and all three DEGs encoding ACC oxidase (Medtr2g025120, Medtr3g088565 and Medtr5g085330), which catalyzes the conversion of ACC into ethylene gas, were expressed at substantially lower levels in RNAi genotypes (Data S2). In contrast, genes involved in abscisic acid (ABA)‐related pathways, including Medtr4g022850 and Medtr4g134870, which encode zeaxanthin epoxidase, Medtr5g025270 and Medtr5g025250, which encode 9‐*cis*‐epoxycarotenoid dioxygenase, and Medtr4g052350, which encodes a short‐chain dehydrogenase/reductase‐like enzyme, demonstrated elevated levels of expression in *MsWOX13‐2* RNAi genotypes compared to wild‐type under waterlogging (Data S2). Similarly, Medtr1g034030, which encodes a sucrose non‐fermenting 1‐related kinase 2 and has been shown to be responsive to both ABA and waterlogging stress previously in alfalfa (Feyissa et al., 2021), was significantly up‐regulated in *MsWOX13‐2* RNAi genotypes compared to wild‐type under waterlogged conditions (Data S1). Likewise, a greater proportion of DEGs associated with gibberellic acid (GA)‐ and salicylic acid (SA)‐related pathways demonstrated elevated expression levels in RNAi genotypes compared to wild‐type under waterlogging (Figure S6d; Data S2). Conversely, DEGs involved in auxin‐, brassinosteroid‐, cytokinin‐, and jasmonate‐related pathways exhibited relatively balanced up‐ and down‐regulation in RNAi genotypes compared to wild‐type (Figure S6d; Data S2).

### WGCNA confirms the involvement of photosynthesis and redox functions in the improved waterlogging resilience of *MsWOX13‐2* RNAi genotypes

To strengthen the mechanistic interpretation of our RNA‐Seq data, we performed a weighted gene co‐expression network analysis (WGCNA). This analysis identified 26 co‐expression modules, each represented by its module eigengene. Module‐trait correlation analysis (Figure S7a) identified several modules that were significantly correlated with genotype and treatment conditions. Notably, the ‘blue’ and ‘midnightblue’ modules exhibited strong, distinct associations with waterlogging and genotype effects. The ‘blue’ module was strongly negatively correlated with waterlogging (correlation coefficient = −0.95, *P* < 1 × 10^−8^), indicating the significant suppression of genes within this module under waterlogging stress. Eigengene expression for this module (Figure S7b) confirmed the general suppression of these genes in response to waterlogged conditions, with *MsWOX13‐2* RNAi genotypes displaying a less severe suppression than the wild‐type genotype. Functional enrichment analysis using GO terms (Data S3) and the identification of the top 30 genes within this module (Data S4) revealed that it was predominantly comprised of genes related to photosynthesis, chloroplast processes, tetrapyrrole biosynthesis, and redox activities, which correlates well with our DEG‐specific GO term (Figure S5) and pathway (Figure 6) analyses.

In contrast, the ‘midnightblue’ module showed a strong positive correlation specifically in *MsWOX13‐2* RNAi genotypes under waterlogging (correlation coefficient = 0.79, *P* < 1 × 10^−4^), suggesting a genotype‐dependent induction. Eigengene analysis confirmed the robust activation of this module exclusively in RNAi genotypes subjected to waterlogging (Figure S7c). GO term enrichment analysis (Data S3) indicated the significant enrichment of nuclear regulatory functions, notably DNA‐binding transcription factor activity, homeobox domain‐containing proteins, chromatin organization, and RNA‐binding processes, which aligned well with the functions of the top 30 genes identified in this module (Data S4). These results suggest that under normal growth conditions, MsWOX13‐2 suppresses (directly or indirectly) the transcription of a suite of regulatory genes.

### Confirmation of waterlogging resilience in *MsWOX13‐2* bi‐allelic alfalfa mutants

Following *Agrobacterium*‐mediated transformation of alfalfa with a CRISPR/Cas9 vector targeting *MsWOX13‐2* (Figure S8a,b), 27 Cas9‐positive T_0_ genotypes were identified. Subsequent droplet digital PCR (ddPCR) evaluation revealed that one independent transformant exhibited a gene editing frequency (GEF) of approximately 25%, which implies the existence of a mutation in one of four *MsWOX13‐2* alleles, and two independent transformants exhibited GEFs of approximately 50%, suggesting the presence of mutations in two of four *MsWOX13‐2* alleles (Figure S8c). All subsequent experiments were carried out with these latter two genotypes (CRISPR‐5 and CRISPR‐11). To precisely identify mutations occurring at the *MsWOX13‐2* guide RNA (gRNA) target site in CRISPR‐5 and CRISPR‐11, we cloned and sequenced this region in these genotypes, along with the untransformed N4.4.2 genotype. As expected, no genetic variation was observed within this region in the untransformed genotype. In contrast, two distinct mutations, both situated 3‐bp upstream of the protospacer adjacent motif (PAM; a 6‐bp deletion and a 24‐bp deletion/1‐bp substitution) were observed along with wild‐type sequence in CRISPR‐5 (Figure S9a). Similarly, two distinct mutations were also identified in CRISPR‐11 (a 16‐bp deletion and a 30‐bp deletion) along with wild‐type sequence (Figure S9a). These results confirm that two of four *MsWOX13‐2* alleles were edited in each of the selected genotypes. Analysis of the predicted amino acid sequences indicated that the 6‐bp deletion and 24‐bp deletion/1‐bp substitution in CRISPR‐5 would result in a 2 amino acid deletion or truncation of the protein by 60 amino acids, respectively (Figure S9b). In the case of CRISPR‐11, deletions of 16‐bp and 30‐bp would lead to protein truncation by 45 and 53 amino acids, respectively (Figure S9b). Sequencing of amplicons spanning the only potential gRNA off‐target site identified indicated that no off‐target mutations were present in either genotype (Table S2). This suggests that the chosen gRNA was highly specific for alfalfa *MsWOX13‐2*.

To determine whether similar improvements in waterlogging resilience were present in the two bi‐allelic CRISPR/Cas9 mutant genotypes, CRISPR‐5 and CRISPR‐11 genotypes, along with empty vector genotypes, were subjected to 3 weeks of waterlogging treatment. As was the case with the *MsWOX13‐2* RNAi genotypes, both edited genotypes exhibited fewer waterlogging‐induced symptoms, such as chlorosis and necrosis, than empty vector genotypes (Figure S10a). Subsequently, the root zone was drained of excess water, and plants were allowed to recover under normal growth conditions for approximately 6 weeks. Following this treatment, plant survival was higher for both edited genotypes (100 and 62.5% survival) compared to empty vector genotypes (41.67% survival; Figure S10b). Taken together, these results support our previous findings with *MsWOX13‐2* RNAi genotypes, indicating that MsWOX13‐2 has a negative influence on waterlogging tolerance in alfalfa.

## DISCUSSION

Waterlogging stress, which is becoming more prevalent due to the effects of climate change (Intergovernmental Panel on Climate Change (IPCC), 2021), can have considerable negative impacts on the growth, yield, and quality of alfalfa (Barta & Sulc, 2002; Castonguay et al., 1993; Striker & Colmer, 2017). However, despite the clear need for improvements in waterlogging tolerance, progress in this area has lagged in this species, at least in part due to the fact that considerable gaps remain in our understanding of general waterlogging response mechanisms in alfalfa. *WOX13* genes, which encode homeodomain‐containing transcription factors, have been shown previously to play a role in abiotic stress response in other plant species, with overexpression in rice and apple leading to enhanced drought tolerance through alterations in the expression of various drought‐responsive genes and an enhancement in enzymatic antioxidant activity, respectively (Lv et al., 2023; Minh‐Thu et al., 2018). Since the function of *WOX13* genes in waterlogging response has yet to be examined in plants, we sought to assess the role of alfalfa MsWOX13‐2 in the context of this type of stress.

*MsWOX13‐2* is one of at least two *MsWOX13* genes in alfalfa, which fall into the ancient clade of the WOX family (Xu et al., 2023; Yang et al., 2022). Interestingly, in the present study, *MsWOX13‐2* was predicted to consist of two exons separated by a single intron. This differed quite substantially from a previous study, where this same gene was suggested to consist of five exons and four introns (Xu et al., 2023), with exons 4 and 5, along with their intervening intron, corresponding to the full *MsWOX13‐2* sequence identified in the current study. Alignment of exon 1 from the Xu et al. (2023) study against the tetraploid alfalfa cv. XinJiangDaYe genome (Chen et al., 2020) suggests that it instead encodes a putative Hap3/NF‐YB family transcription factor. This gene is located approximately 7.2‐kb upstream of the *MsWOX13‐2* transcriptional start site predicted in the current study. Exons 2 and 3 from the Xu et al. (2023) *MsWOX13‐2* sequence, in contrast, appear to be located in an intergenic region between the putative Hap3/NF‐YB family gene (Chen et al., 2020) and the *MsWOX13‐2* gene in the current study. The genetic structure suggested in the present study closely resembles that of the *M. truncatula WOX13‐2* homolog and is also consistent with gene annotations provided in various tetraploid (Chen et al., 2020; Long et al., 2022) and diploid (Shen et al., 2020) *M. sativa* genome sequences. As such, we have adopted the two‐exon, one‐intron structure for this study (Figure S1a).

In terms of tissue specificity, we found that *MsWOX13‐2* transcript abundance was relatively high in roots (excluding nodules), with somewhat lower levels of expression in leaves, stems, and flowers, and significantly lower levels in nodules (Figure 1A). These results contrast with previous findings, whereby the corresponding gene was found to be expressed predominantly in nodules in a rhizobia‐responsive manner, with intermediate levels of expression in stem tissue (Xu et al., 2023); a discrepancy that could feasibly be due to differences in the *MsWOX13‐2* sequence, alfalfa genotypes assessed, growth conditions, and/or the particular tissues evaluated in each study. Our expression results were corroborated by our finding that the substantial down‐regulation of *MsWOX13‐2* in RNAi genotypes (up to 17‐fold reduction in transcript levels compared to wild‐type) did not have any impact on the production of healthy nodules or the ability of plants to grow in the absence of supplemental nitrogen when inoculated with rhizobia (Figure S2a–f).

We also determined that the expression of *MsWOX13‐2* in leaf tissues was responsive to waterlogging stress, with transcript levels being significantly down‐regulated by the third week of treatment (Figure 1B). In line with its waterlogging responsiveness, we subsequently observed that the RNAi‐mediated silencing and CRISPR/Cas9‐mediated bi‐allelic mutation of *MsWOX13‐2* enhanced capacity to withstand waterlogging stress in alfalfa, which was evidenced by reduced leaf senescence following waterlogging treatment and superior survivability after subsequent re‐oxygenation (Figure 2; Figure S10). In addition, although no significant differences were noted between the shoot and root morphology or growth of *MsWOX13‐2* RNAi and empty vector genotypes under normally watered conditions, both *MsWOX13‐2* RNAi genotypes exhibited less shoot growth and root length inhibition following 2 weeks of waterlogging (Figure 3A–E), which is of particular importance in forage crops such as alfalfa.

At least certain plant species/genotypes develop aerenchyma, which are gas cavities formed through the death of cortical cells as a means of improving oxygen diffusion in their roots as an adaptive mechanism under waterlogging stress (Leeggangers et al., 2023). However, no aerenchyma were produced in any genotype following waterlogging in the present study (Figure S3). Although aerenchyma have been observed in alfalfa in response to waterlogging previously (Zook et al., 1986), this has not always been the case (Teutsch & Sulc, 1997), and it is currently unclear how prevalent this mechanism is in this species. In any case, our results suggest that alterations in aerenchyma production did not contribute to the waterlogging tolerance observed in *MsWOX13‐2* RNAi genotypes.

Chlorophyll degradation and the inhibition of photosynthesis are typical physiological symptoms of waterlogging stress in the aboveground tissues of plants, including alfalfa (Feyissa et al., 2021; Zeng et al., 2019; Zhang et al., 2019). In line with this, all alfalfa genotypes assessed in the current study exhibited some level of leaf chlorosis and reductions in chlorophyll content, as well as declines in various photosynthesis‐related characteristics following waterlogging treatment (Figures 2A and 4). However, in the case of chlorophyll content, *A*_sat_, and *F*_v_/*F*_m_, these reductions were significantly less severe in *MsWOX13‐2* RNAi than in empty vector genotypes (Figure 4A,C,G). Correspondingly, GO term enrichment analysis of RNA‐Seq data derived from the leaves of alfalfa subjected to waterlogging stress indicated a significant enrichment of genes associated with photosynthesis‐related functions in *MsWOX13‐2* RNAi genotypes compared to wild‐type following waterlogging treatment (Figure S5b; Files S3 and S4), and the vast majority of DEGs associated with photosynthesis were significantly up‐regulated in RNAi genotypes compared to the wild‐type (Figure 6B; Data S2). Overall, these physiological and transcriptomic responses in *MsWOX13‐2* RNAi genotypes following waterlogging treatment suggest that the suppression of *MsWOX13‐2* may be associated with the improved maintenance of photosynthetic function during waterlogging stress.

Waterlogging stress is also known to substantially augment the accumulation of ROS within plant cells (e.g., Ahsan et al., 2007; Sairam et al., 2009), and when at high enough levels, these ROS damage cell membranes, proteins, and nucleic acids (Hasanuzzaman et al., 2020). In the present study, we noted a tendency for higher H_2_O_2_ accumulation in the leaves of empty vector genotypes under waterlogged conditions (Figure 5A). This corresponded with a significant increase in the accumulation of MDA, which acts as a biomarker of lipid peroxidation (Hodges et al., 1999), in empty vector genotypes under waterlogged compared to normally watered conditions (Figure 5B). Interestingly, this same phenomenon was not observed in *MsWOX13‐2* RNAi genotypes (Figure 5A,B). These results suggest that the *MsWOX13‐2* RNAi genotypes experienced less oxidative damage when exposed to hypoxic/anoxic conditions, which is typical in waterlogging‐tolerant plants (e.g., Shah et al., 2024). While it is possible that the *MsWOX13‐2* RNAi genotypes accumulated lower levels of ROS due to the fact that they were less impacted by waterlogging treatment, it is also feasible that these genotypes were better able to scavenge ROS through the enhanced activation of antioxidant systems, which could occur via alterations in the activity/levels of various enzymatic and non‐enzymatic antioxidants.

In terms of enzymatic antioxidant activity, we found that the activity of SOD, which transforms O_2_^−^ into H_2_O_2_ and is the first line of defense against ROS (Szőllősi, 2014), increased significantly between normally watered and waterlogged conditions in the leaves of *MsWOX13‐2* RNAi genotypes, but not empty vector genotypes (Figure 5C). This finding suggests that the suppression of *MsWOX13‐2* enhances alfalfa's ability to dismutate O_2_^−^. In line with this, we also observed the significant up‐regulation of several SOD‐encoding genes, including Medtr1g048990 (SOD [Fe]), Medtr3g094250 (SOD [Mn]), and Medtr4g057240 (SOD [Cu–Zn]), in our RNAi genotypes compared to wild‐type under waterlogged conditions (Figure S6b; Data S2). Although we found a reduction in H_2_O_2_ accumulation in *MsWOX13‐2* RNAi genotypes compared to empty vector genotypes under waterlogging stress (Figure 5A), we did not observe an increase in the activities of APX or CAT, which function in the scavenging of H_2_O_2_ (Anjum et al., 2016), in *MsWOX13‐2* RNAi genotypes compared to wild‐type (Figure 5D,E). This implies that other antioxidants could be of importance in the neutralization of H_2_O_2_ in *MsWOX13‐2* RNAi genotypes. Intriguingly, a number of genes encoding peroxiredoxins, which also play a key role in H_2_O_2_ detoxification (Bhatt & Tripathi, 2011), displayed elevated transcript levels in RNAi genotypes compared to wild‐type under waterlogging stress (Figure S6b; Data S2), which could contribute to the observed reduction in H_2_O_2_ in these plants.

Non‐enzymatic antioxidants, including flavonoids such as anthocyanin and flavonol, are also effective in ROS scavenging in plants and alleviate cellular damage incurred from oxidative damage (Garcia‐Caparros et al., 2021). Previous studies have shown that leaf flavonoid levels can correlate positively with waterlogging tolerance (e.g., Olorunwa et al., 2022; Shah et al., 2024). However, in the present study, while both anthocyanin and flavonol indices increased significantly in response to waterlogging stress in the leaves of *MsWOX13‐2* RNAi and empty vector genotypes (Figure 5G,H), empty vector genotypes accumulated significantly higher levels of anthocyanin than *MsWOX13‐2* RNAi genotypes under waterlogging (Figure 5F,G). This latter finding could be related to the fact that the RNAi genotypes were not as severely stressed under waterlogged conditions; a phenomenon that has been observed previously in certain waterlogging‐tolerant plants (Savchenko et al., 2019). In line with these results, DEGs encoding CHS enzymes, which are key and rate‐limiting enzymes in the flavonoid biosynthetic pathway (Liu et al., 2021), exhibited significant down‐regulation in *MsWOX13‐2* RNAi genotypes compared to wild‐type plants under waterlogging (Data S2). This suggests that although an increase in flavonoid production may play a role in tolerance to this type of stress in certain instances (e.g., Feyissa et al., 2021), a different mechanism drives this response in *MsWOX13‐2* RNAi genotypes. In contrast, a large proportion of genes involved in terpene metabolism were transcribed at higher levels in *MsWOX13‐2* RNAi genotypes compared to wild‐type under waterlogging (Figure 6B; Data S2). Given the antioxidant properties of terpenes (Sinha et al., 2024), along with the previously documented up‐regulation of waterlogging‐induced terpene biosynthetic genes in tolerant genotypes of other crop species (e.g., Bai et al., 2024; Sun et al., 2023), it is possible that terpenes might play a role in the waterlogging tolerance observed in *MsWOX13‐2* RNAi genotypes.

Proline is another compound with potential antioxidant, as well as osmoprotectant, functions (Ghosh et al., 2022). Correspondingly, its increased accumulation has, at least in certain instances, been correlated with waterlogging tolerance in plants (e.g., Habibullah et al., 2021). However, this is certainly not always the case (e.g., Arbona et al., 2008), and in the current study, we found that unlike empty vector genotypes, *MsWOX13‐2* RNAi genotypes did not exhibit enhanced proline accumulation in leaves following waterlogging treatment (Figure 5I). These findings suggest that proline accumulation might be more indicative as a symptom, rather than a response, to waterlogging in alfalfa, which is akin to findings in various other crop species in the context of drought stress (e.g., Hang et al., 2021; Wang et al., 2017). Soluble carbohydrates, on the contrary, tend to play a more multifaceted role than proline, acting as metabolic fuel, signaling molecules, and osmolytes to protect cells from damage (Saddhe et al., 2021). In waterlogging‐sensitive genotypes/species, soluble carbohydrate levels tend to increase following waterlogging stress, possibly due to an imbalance in the unloading of sugars in waterlogged roots, which then leads to the increased accumulation of sugars in shoots (Daugherty & Musgrave, 1994; Lothier et al., 2020). In the current study, while significant increases in soluble carbohydrates were noted in the leaves of empty vector genotypes under waterlogging, this was not consistently the case in *MsWOX13*‐2 RNAi genotypes (Figure 5J), which could feasibly be due to improved unloading of carbohydrates in their roots.

Alterations in energy production mechanisms, including the metabolism of carbohydrates through glycolytic and fermentative pathways to generate ATP and sustain the generation of energy in the absence of oxygen, also tend to be a major factor affecting the survival of waterlogged plants (Pan et al., 2021). The fermentation pathway primarily encompasses the utilization of pyruvate produced from glycolysis via lactic acid and ethanolic fermentation; the former of which involves the conversion of pyruvate to lactic acid through the catalytic activity of LDH, while the latter entails the conversion of pyruvate to acetaldehyde through the catalytic activity of PDC followed by reduction to ethanol through the activity of ADH (Pan et al., 2021). In the present study, the majority of DEGs encoding enzymes that function in glycolysis were up‐regulated in the leaves of *MsWOX13‐2* RNAi genotypes compared to wild‐type under waterlogging, which suggests an enhancement in glycolysis for energy production in RNAi genotypes following this type of stress (Figure 6B; Data S2). We also observed the up‐regulation of DEGs encoding LDH and ADH in *MsWOX13‐2* RNAi genotypes compared to wild‐type under waterlogging stress (Data S2). While the increased expression/activity of these genes/enzymes has, at least in certain instances, been associated with improvements in waterlogging tolerance previously (e.g., Bertolde et al., 2014; Chiang et al., 2017; Yin et al., 2019), this is not always the case (e.g., Bertolde et al., 2014; Yin et al., 2019), and further research will be required to unravel their effects on the waterlogging tolerance observed in *MsWOX13‐2* RNAi genotypes.

The roles of phytohormones have also been well‐studied in various plant species in the context of waterlogging stress (e.g., Feyissa et al., 2021; Pan et al., 2021; Wang & Komatsu, 2022). In the current study, genes functioning in pathways related to several phytohormones, including ethylene, ABA, auxin, cytokinin, GA, SA, brassinosteroids, and jasmonates, were transcriptionally altered in *MsWOX13‐2* RNAi genotypes compared to wild‐type under waterlogged conditions (Figure S6d; Data S2). Ethylene, in particular, has been shown to play an important role in waterlogging response/tolerance (Khan et al., 2020). Generally, when plants sense hypoxia, there is an abundant conversion of SAM to ACC through the catalytic activity of acetyl‐CoA synthetase (ACS), which is then converted into ethylene via the activity of 1‐aminocyclopropane‐1‐carboxylic acid oxidase (ACO) in roots and shoots (Houben & Van de Poel, 2019; Leeggangers et al., 2023). In the present study, we observed the significant down‐regulation of genes encoding both ACS and ACO in the leaves of waterlogged *MsWOX13‐2* RNAi genotypes compared to wild‐type, suggesting that the RNAi genotypes may curtail ethylene production compared to wild‐type plants under this type of stress (Data S2). This corresponds with previous findings in tomato, whereby SlWOX13 has been shown to positively regulate the expression of various genes involved in ethylene biosynthesis and signaling, at least in some cases through the direct binding of their promoters (Jiang et al., 2024). While it has been suggested previously that ethylene accumulation in leaf tissues may be associated with waterlogging adaptation in various plant species (e.g., Cheng et al., 2024; Geng et al., 2023), its excessive production and/or the constitutive activation of ethylene signaling has also been linked to detrimental impacts such as dwarfing and premature senescence (Chao et al., 1997; Dubois et al., 2018). As such, it is plausible that the wild‐type alfalfa genotype utilized in the current study may produce an excess of ethylene under waterlogged conditions, thus contributing to its phenotypic decline in response to this type of stress. In any case, further study, including ethylene quantification and the identification of direct MsWOX13 targets, will be a necessity to confirm this theory.

ABA is another phytohormone with multifaceted roles that has been less well‐studied than ethylene in the context of waterlogging, but has been suggested to facilitate waterlogging adaptation through the induction of stomatal closure and the delay of leaf senescence/cell death, for example (Wang & Komatsu, 2022). In the current study, five of seven DEGs related to ABA biosynthesis, as well as a gene encoding SRK2, which was previously found to be responsive to waterlogging in an ABA‐dependent manner in alfalfa (Feyissa et al., 2021), were up‐regulated in the leaves of *MsWOX13‐2* RNAi genotypes compared to wild‐type under waterlogged conditions (Figure S6d; Data S1 and S2). These results indicate a possible function for ABA in terms of conferring superior waterlogging tolerance in the RNAi genotypes. However, there have been conflicting results among studies in terms of the transcriptional regulation of ABA biosynthetic genes in the leaves of hypoxia‐tolerant genotypes compared to susceptible genotypes (e.g., Feyissa et al., 2021; Gedam et al., 2023; Xu et al., 2017), and these changes have not always corresponded with alterations in ABA levels in alfalfa (e.g., Feyissa et al., 2021). As such, the downstream evaluation of ABA content will be a necessity to determine the possible contribution of ABA in the superior waterlogging tolerance of *MsWOX13‐2* RNAi alfalfa genotypes.

Transcription factors also provide crucial roles in the response of plants to various abiotic stresses, including waterlogging, by modulating the transcription of downstream genes involved in a plethora of processes (Yang et al., 2023). In the present study, several DEGs encoding members of WRKY, bZIP, NAC, PHOR1, bHLH, AP2‐EREBP, HB, Aux/IAA, and ARF families, which in many cases have been reported to be involved in waterlogging tolerance in plant species previously (e.g., Raineri et al., 2022; Wang et al., 2021; Wei et al., 2019; Xing et al., 2024), were differentially expressed between the leaves of *MsWOX13‐2* RNAi and wild‐type genotypes under waterlogging stress (Figure S6c; Data S2). Notably, a gene encoding an AP2 domain class transcription factor (Medtr3g098580) was down‐regulated in *MsWOX13‐2* RNAi genotypes compared to wild‐type under waterlogged conditions, which was also observed in other waterlogging‐tolerant alfalfa genotypes in a previous study (Feyissa et al., 2021), implying a possible common tolerance mechanism in this species.

**Figure 7:**
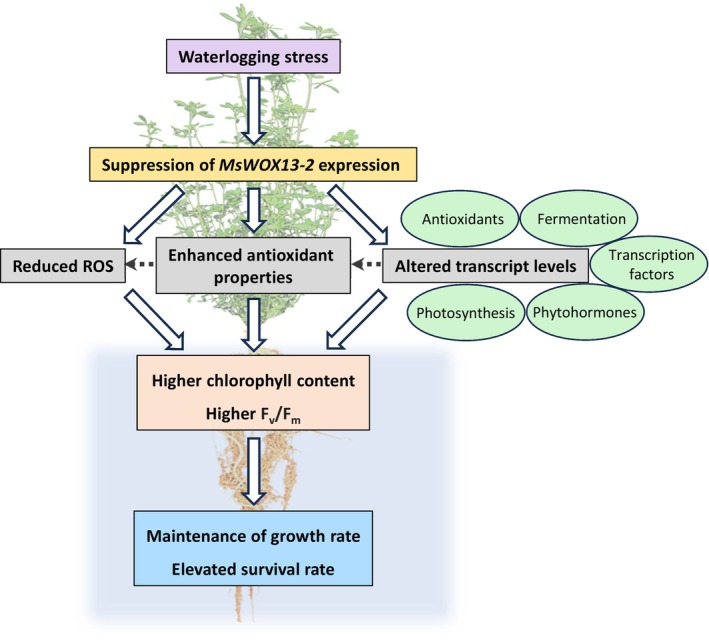
Proposed model illustrating the role of MsWOX13‐2 in alfalfa under waterlogging stress. The transcriptional suppression of *MsWOX13‐2* under waterlogging results in decreased levels of ROS, increased antioxidant activity, and altered transcript levels of genes associated with antioxidants, photosynthesis, fermentation, phytohormones, and transcription factors. This, in turn, leads to higher chlorophyll content and improved maximum quantum efficiency of photosystem II (*F*_v_/*F*_m_) in alfalfa leaves, supporting sustained growth and enhanced survival under stress conditions. The dotted arrow indicates a putative indirect linkage.

In conclusion, we functionally characterized *MsWOX13‐2* in alfalfa and found this gene to be transcriptionally responsive to waterlogging in leaves. We also found that while alfalfa genotypes with reduced *MsWOX13‐2* expression levels did not differ significantly from wild‐type/empty vector plants in terms of nodulation, growth, or morphological characteristics under normally watered conditions, they performed better under waterlogging stress. This reduction in stress symptoms could be attributed, at least in part, to the fact that the leaves of *MsWOX13‐2* RNAi genotypes possessed enhanced SOD activity, reduced H_2_O_2_ accumulation and oxidative damage, and altered transcript levels of genes associated with antioxidants, photosynthesis, fermentation, phytohormones, and transcription factors (Figure 7). Although transcriptomic alterations were not assessed in the roots of *MsWOX13‐2* RNAi genotypes in the current study, tissue‐specific divergence in *WOX13* gene function has been observed previously in other plant species (Cheng et al., 2023; Deveaux et al., 2008; Li & Zhang, 2025; Minh‐Thu et al., 2018), suggesting that MsWOX13‐2 could also regulate distinct root‐specific targets. As such, further research will be required to unravel the precise mechanisms driving the function of MsWOX13‐2, particularly in roots. In any case, our findings suggest a negative regulatory role for MsWOX13‐2 in the waterlogging stress response of alfalfa and provide a potential target gene for future breeding efforts in this important forage crop.

## MATERIALS AND METHODS

### Plant material, growth conditions, and stress treatment

Plants were grown in a greenhouse maintained at approximately 20°C/15°C day/night temperatures, with supplemental lighting providing a 16/8 h photoperiod and a light intensity of approximately 150 μmol m^−2^ sec^−1^. Unless otherwise indicated, plants were grown in 10.5 cm (length) × 10.5 cm (width) × 12.5 cm (height) pots filled with Cornell soilless potting mix (Boodley & Sheldrake Jr., 1972). With the exception of nodulation studies, plants were not inoculated with rhizobia. Due to the obligatory outcrossing nature of alfalfa, all genotypes were propagated vegetatively via stem cuttings throughout the study to maintain particular genotypes and provide pools of biological replicate plants (the precise number of biological replicates used in each experiment is specified in the relevant figure legends). In all cases, 3‐week‐old rooted stem cuttings of similar size were transferred to pots at the same time point and were cut back 2–4 times to approximately 5 cm in height, unless otherwise specified, prior to assessments. The N4.4.2 alfalfa genotype (Badhan et al., 2014) was used as wild‐type throughout the study, while three independent phenotypically normal empty vector genotypes (based on the N4.4.2 genotype) were used as controls in growth, photosynthetic, and biochemical assessments.

Waterlogging stress was imposed by submerging and maintaining pots in water to 3/4 of pot height. All morphological, physiological, and biochemical analyses were carried out 7 and/or 14 days following the initiation of treatment, when wild‐type alfalfa had begun to display obvious stress symptoms such as chlorosis. Survivability after exposure to waterlogging stress and subsequent re‐oxygenation, which contributes to further oxidative stress (Jethva et al., 2022), was resolved by calculating the percentage of plants that survived from each group after 14 days (RNAi genotypes) or 21 days (edited genotypes) of waterlogging exposure, followed by drainage and 6 weeks of growth under normally watered conditions.

### Evaluation of the tissue‐specificity and waterlogging responsiveness of *MsWOX13‐2*

The sequence of all four *MsWOX13‐2* alleles was obtained via a Basic Local Alignment Search Tool search of a tetraploid alfalfa genome (cv. XinJiangDaYe; Chen et al., 2020) using the *M. truncatula* Medtr7g023810 sequence as a query. The position of the homeodomain within the predicted MsWOX13‐2 amino acid sequence was determined using SMART (http://smart.embl.de/; accessed November 19, 2024).

In order to assess the expression levels of *MsWOX13‐2* in different tissues, samples were harvested from biological replicate plants of the wild‐type genotype. In the case of leaves, middle leaflets from first fully expanded trifoliate leaves (closest to the shoot tip) were used. Stem tissue comprised the region between the fourth and fifth nodes from the shoot tip. Flowers consisted of whole open flowers, roots comprised lateral roots near the root tips, and nodules included fully grown, pink nodules. For the assessment of waterlogging responsiveness, middle leaflets from fully expanded trifoliate leaves (first from the shoot tip) were harvested from wild‐type alfalfa 1, 7, 14, and 21 days after the initiation of waterlogging stress, as well as at the same time points under normally watered conditions. All samples were flash frozen in liquid nitrogen and stored at −80°C until processed.

Total RNA was extracted from all samples using the Spectrum Plant Total RNA kit with on‐column DNAse treatment following the manufacturer's instructions (Sigma‐Aldrich Corp., St. Louis, MO, USA). RNA quality was assessed on a 1% agarose gel, and concentrations were determined using a NanoDrop 8000 spectrophotometer (Thermo Fisher Scientific Inc., Waltham, MA, USA). First‐strand cDNA was generated using the Superscript IV cDNA synthesis kit (Thermo Fisher Scientific) with 2 μg RNA as template and oligodT primer in a final volume of 20 μl.

qRT‐PCR assays were performed using undiluted cDNA template along with primers MsWOX13‐2qPCRF1 and MsWOX13‐2qPCRR1, which were designed to anneal to a region of the *MsWOX13‐2* coding sequence (Table S3), and PerfeCTa SYBR Green Supermix (Quantabio, Beverly, MA, USA) in a final volume of 10 μl. The *ACTIN DEPOLYMERIZING FACTOR* (*ADF*) gene, which has been found to be stably expressed in alfalfa under various abiotic stress conditions and tissue types previously, was used as an internal control for normalization using primers MsADFF1 and MsADFR1 (Castonguay et al., 2015; Table S3). Three technical replicates were included for each sample. Assays were carried out using a QuantStudio 6 Flex Real‐Time PCR system (Thermo Fisher Scientific) using the following thermal parameters: initial denaturation at 95°C for 2 min, followed by 40 cycles of 95°C for 15 sec and 55°C for 30 sec. Melting curves were produced to confirm the presence of a single amplification product in each case, and gene expression levels were determined using the comparative ∆∆ cycle threshold method (Livak & Schmittgen, 2001).

### Vector construction and *Agrobacterium*‐mediated transformation

Details regarding the construction of the binary RNAi vector can be found in Methods S1. The *MsWOX13‐2* RNAi and *MsWOX13‐2* CRISPR vectors, as well as the respective empty vectors, were introduced into the wild‐type N4.4.2 alfalfa genotype via the *Agrobacterium*‐mediated transformation of leaf explants. Transformed plantlets were validated using PCR, as described previously (Singer et al., 2022).

### Validation of *MsWOX13‐2* down‐regulation in RNAi genotypes

Total RNA was extracted from middle leaflets (first fully expanded leaflet from the shoot tip) as described in a previous section, and subjected to an additional DNAse treatment using the Turbo DNA‐free kit (Thermo Fisher Scientific). First‐strand cDNA synthesis and qRT‐PCR assays were performed as described in a previous section, using primers WOX13‐2qPCRF1 and WOX13‐2qPCRR1 for amplification of the *MsWOX13‐2* fragment, and MsADFF1 and MsADFR1 for amplification of an *ADF* fragment as the internal control (Table S3). The 2 independent *MsWOX13‐2* RNAi genotypes with the greatest levels of transcriptional down‐regulation were selected for all downstream evaluations (termed RNAi‐203 and RNAi‐205).

To confirm the specificity of the *MsWOX13‐2* RNAi vector used in this study, we also assessed *MsWOX13‐1* transcript levels in our selected RNAi genotypes. qRT‐PCR assays were performed as described above, using cDNA template (diluted 1:50) from RNAi‐203 and RNAi‐205 genotypes, as well as wild‐type, along with primers WOX13‐1qPCRF1 and WOX13‐1qPCRR1 (Table S3).

### Confirmation of CRISPR/Cas9‐mediated gene editing

GEFs of transgenic alfalfa genotypes bearing CRISPR/Cas9 cassettes were assessed using GEF‐ddPCR as described previously (Singer et al., 2022; Subedi et al., 2023; Table S3). To examine the precise nature of mutations at the gRNA target site, a 473‐bp region of *MsWOX13‐2* (encompassing the gRNA target site) was amplified from wild‐type and two edited genotypes (derived from independent transformation events) exhibiting a GEF of at least 50% using primers MsWOX13geneseqF1 and MsWOX13geneseqR1 (Table S3) and the high‐fidelity Platinum SuperFi Green PCR Master Mix (Thermo Fisher Scientific) with a thermal program of 98°C for 30 sec, 35 cycles of 98°C for 10 sec, 57°C for 10 sec, and 72°C for 30 sec, and a final extension at 72°C for 1 min. The resulting amplicons were then cloned into the pGEM‐T Easy cloning vector (Promega Corp., Madison, WI, USA) and 24 clones from each genotype were sequenced (Bio Basic Inc., Markham, ON, Canada). Off‐target analysis of the only identified potential off‐target site in wild‐type and the two edited genotypes was carried out as described previously (Singer et al., 2022) using primers WOX13gRNAoffF1 and WOX13gRNAoffR1 (Table S3). Five to eight clones were sequenced in each case.

### Growth and morphological evaluations

The assessment of various morphological parameters under both normally watered and waterlogged conditions was conducted approximately 2 weeks after the initiation of waterlogging stress (all plants were pre‐flowering at this time point). Plant height was measured as the length of the tallest shoot, while the number of primary branches was assessed by counting the total number of primary branches on each plant. To determine shoot fresh weight, all aboveground biomass was harvested and immediately weighed, while shoot dry weight was resolved by drying tissue at 65°C for 1 week prior to weighing. For the evaluation of root parameters, roots were washed thoroughly, and root length was determined as the length of the longest primary root, while root dry weight was evaluated by drying roots at 65°C for 1 week before weighing.

Nodulation and nitrogen fixation capacity were assessed in plants grown under normally watered conditions as described in Methods S1. Root aerenchyma formation was evaluated under normally watered growth conditions, as well as following 14 and 28 days of waterlogging, as described in Methods S1.

### Determination of chlorophyll and photosynthetic parameters

Chlorophyll content was assessed in middle leaflets from fully expanded trifoliate leaves (first from the shoot tip) using a CCM‐300 Chlorophyll Content Meter (Opti‐Sciences, Hudson, NH, USA). Two to three leaflets from each biological replicate were evaluated. *E*, *A*_sat_, *g*_sw_, ETR, *Φ*_PSII_, and *F*_v_/*F*_m_ were determined using a LI‐6800 system (LI‐COR Inc., Lincoln, NE, USA) and middle leaflets of fully expanded trifoliate leaves (third from the shoot tip) from plants grown under normally watered conditions and following 7 and 14 days of waterlogging as described previously (Singer et al., 2023), with the exception of the chamber temperature, which was set to 23°C.

### Evaluation of lipid peroxidation and ROS

MDA levels were determined using the QuantiChrom TBARS Assay Kit according to the manufacturer's instructions (BioAssay Systems, Hayward, CA, USA). Middle leaflets from fully expanded trifoliate leaves (first from the shoot tip) from plants grown under waterlogged and normally watered conditions were harvested 14 days after the initiation of treatment, then flash frozen and freeze dried for 24 h prior to assessment.

A histochemical staining procedure was used to detect the accumulation of H_2_O_2_ in alfalfa leaves using DAB as described previously (Singer et al., 2023). Three middle leaflets from fully expanded trifoliate leaves (fourth from the shoot tip) were evaluated from plants grown under both normally watered and waterlogged conditions 14 days after the initiation of treatment.

### Determination of antioxidant‐related characteristics and osmolyte levels

Enzymatic antioxidant activities were assessed using fully expanded trifoliate leaves (third from the shoot tip) from plants grown under normally watered and waterlogged conditions 10 days following the initiation of treatment as described previously (Singer et al., 2021). Anthocyanin and flavonol indices were evaluated in two middle leaflets from fully expanded trifoliate leaves (fifth from the shoot tip) from each plant grown under normally watered and waterlogged conditions 15 days after the initiation of treatment. Measurements were carried out using the abaxial side of each leaflet and an MPM‐100 Multi Pigment Meter (ADC BioScientific Ltd., Hoddesdon, UK).

In the case of osmolyte‐related assessments, middle leaflets from fully expanded trifoliate leaves (third from the shoot tip) were harvested 14 days after the initiation of waterlogging or normally watered treatment, flash frozen in liquid nitrogen, and then freeze dried for 24 h. Proline was quantified according to Bates et al. (1973) with minor modifications as described previously (Singer et al., 2023). Total soluble carbohydrate content was assessed using the Plant Soluble Sugar Content Assay Kit according to the manufacturer's instructions (MyBioSource Inc., San Diego, CA, USA).

### RNA‐Seq analysis

Middle leaflets from fully expanded trifoliate leaves (first from the shoot tip) were harvested from three biological replicate plants of the wild‐type genotype, as well as RNAi‐203 and RNAi‐205 genotypes, respectively, which had been grown under normally wateredand waterlogged conditions for 14 days. Leaflets were flash frozen in liquid nitrogen and stored at −80°C until total RNA was extracted as described in a previous section. Quality control, library preparation, sequencing, mapping of sequencing reads, differential gene expression analysis, and GO term enrichment analysis were all carried out by Novogene Corporation Inc. (Sacramento, CA, USA), with detailed protocols provided in Methods S1. Significant DEGs were analyzed and mapped into several pathways using MapMan v3.6 (https://mapman.gabipd.org/; accessed April 15, 2023), with the *M. truncatula* genome (Mt4.0 v2) as a reference. WGCNA was performed using the WGCNA R package (Langfelder & Horvath, 2008) as described in Methods S1.

### qRT‐PCR validation of RNA‐Seq results

For RNA‐Seq validation, 10 significantly up‐ or down‐regulated DEGs related to stress response were selected for qRT‐PCR (Table S4). First‐strand cDNA synthesis was performed using 1 μg RNA (from the same samples utilized for RNA‐Seq analysis), the Superscript VILO cDNA synthesis kit (Thermo Fisher Scientific), and oligodT primer in a final volume of 20 μl. qRT‐PCR assays were carried out in triplicate as described in previous sections, using primers designed to specifically amplify regions of each selected gene (Table S4), with *ADF* as an internal control for normalization. Relative expression levels were determined using the comparative ∆∆ threshold cycle method and were converted into log_2_ fold‐changes. The correlation coefficient was then resolved as a means of comparing qRT‐PCR and RNA‐Seq data.

### Statistical analyses

All experimental data were initially subjected to normality evaluations using the Shapiro–Wilk test before proceeding with further analyses. Upon passing the criteria, data requiring comparisons of multiple groups were subjected to either one‐way or two‐way analysis of variance (anova) assessments, followed by *post‐hoc* pairwise comparisons among groups using the Tukey–Kramer honestly significant difference test (Tukey HSD, *P* ≤ 0.05). When the normality assumption failed, data were compared using the Kruskal–Wallis test followed by Dunn's multiple comparison test with ‘Bonferroni’ adjustment. All analyses were carried out in R using RStudio software (RStudio Team 2023, Boston, MA, USA). Two‐tailed *t*‐tests (assuming unequal variance) were used to assess differences between the means of paired datasets using Microsoft Excel 365 (Microsoft Inc., Redmond, WA, USA).

## AUTHOR CONTRIBUTIONS

SDS, AH, SA, and GC conceived and designed the experiments; SDS and SA acquired funding for the project; US, ML, and KBH performed morphological and physiological experiments; US and CVN carried out molecular, biochemical, and RNA‐Seq‐related experiments; US and CVN analyzed the data; GC and SDS were responsible for the supervision of students and technicians; US and SDS wrote the manuscript. All authors reviewed and approved the final manuscript.

## CONFLICT OF INTEREST STATEMENT

The authors declare no competing interests.

## Supporting information


**Methods S1.** Supplementary materials and methods.

**Data S1.** List of differentially expressed genes (DEGs) between wild‐type and *MsWOX13‐2* RNAi genotypes grown under normally watered (C) and waterlogged (W) conditions, respectively, and between normally watered (C) and waterlogged (W) conditions in *MsWOX13‐2* RNAi and wild‐type genotypes, respectively.

**Data S2.** Information regarding differentially expressed genes (DEGs) between wild‐type and *MsWOX13‐2* RNAi genotypes under waterlogging stress falling into a selection of MapMan categories with putative functions in abiotic stress response.

**Data S3.** GO term enrichment analysis of ‘blue’ and ‘midnight blue’ WGCNA modules.

**Data S4.** List of top 30 hub genes in ‘blue’ and ‘midnight blue’ WGCNA modules.

**Figure S1.** Gene structure, vectors, and assessment of *MsWOX13‐2* and *MsWOX13‐1* transcript levels in *MsWOX13‐2* RNAi genotypes.
**Figure S2.** Impact of *MsWOX13‐2* down‐regulation on nodulation in alfalfa.
**Figure S3.** Root cross‐sections for the visualization of aerenchyma in *MsWOX13‐2* RNAi and wild‐type genotypes under normally watered conditions and after 14 and 28 days of waterlogging.
**Figure S4.** Analysis of differentially expressed genes (DEGs) in wild‐type and *MsWOX13‐2* RNAi leaf tissues under normally watered conditions and after waterlogging for 14 days.
**Figure S5.** Gene Ontology (GO) term enrichment analysis of differentially expressed genes (DEGs) between wild‐type and *MsWOX13‐2* RNAi genotypes under normally watered (a) and waterlogged (b) conditions.
**Figure S6.** Transcriptional alteration of genes involved in abiotic stress‐related pathways, redox, transcription factor families, and phytohormonal regulation in *MsWOX13‐2* RNAi genotypes compared to wild‐type under waterlogging stress.
**Figure S7.** Weighted gene co‐expression network analysis (WGCNA) of RNA‐Seq data from *MsWOX13‐2* RNAi and wild‐type genotypes under normally watered and waterlogged conditions.
**Figure S8.** CRISPR/Cas9‐mediated gene editing of *MsWOX13‐2* in alfalfa.
**Figure S9.** Identification of *MsWOX13‐2* edits and confirmation of a lack of off‐target mutations.
**Figure S10.** Waterlogging resilience of *MsWOX13‐2* CRISPR genotypes compared to empty vector genotypes.

**Table S1.** Summary of Illumina sequencing data and mapped reads for each sample.
**Table S2.** Off‐target mutation analysis.
**Table S3.** Primers and probes used in vector construction, qRT‐PCR assessments of *MsWOX13* transcript levels, and the analysis of edited genotypes.
**Table S4.** List of primers used for the qRT‐PCR validation of RNA‐Seq results.

## Data Availability

The RNA‐Seq data generated in this study are available at the National Center for Biotechnology Information (NCBI) Sequence Read Archive (BioProject accession number PRJNA1193595).
