# Supplementary material for: The down‐regulation of MsWOX13‐2 promotes enhanced waterlogging resilience in alfalfa
Source: Plant J. 2025 Aug 20;123(4):e70411. doi: 10.1111/tpj.70411 (PMC12368323; doi:10.1111/tpj.70411)
Supplement: Supplementary file 1 — Methods S1. Supplementary materials and methods. [file TPJ-123-0-s005.docx]

**The down-regulation of *MsWOX13-2* promotes enhanced waterlogging resilience in alfalfa**

**Supporting Methods**

*Generation of MsWOX13-2 RNAi and CRISPR vectors*

To generate the *MsWOX13-2* RNAi vector, primers were designed with appropriate restriction sites at their 5’ ends (Table S1) to amplify 319 bp fragments (both sense and antisense orientations) of *MsWOX13-2* near the 3’ terminus of the coding sequence from cDNA derived from the wild-type N4.4.2 alfalfa genotype. The specificity of the RNAi fragment was confirmed by screening a tetraploid alfalfa genome database (Chen et al., 2020) using BLAST with Geneious Prime software (Biomatters Inc., San Diego, CA, United States). PCR amplification was carried out using the high fidelity Platinum SuperFi Green PCR Master Mix (Thermo Fisher Scientific) and 2 µL of cDNA in a final volume of 20 µL. Thermal cycling conditions included an initial denaturation at 98°C for 30 s, 35 cycles of 98°C for 10 s, 55°C for 10 s, and 72°C for 30 s, and a final extension at 72°C for 5 min.

A binary background RNAi vector was generated by first replacing the existing cassette between the left and right T-DNA borders of the pANIC 6A vector (Mann et al., 2012) with a selection cassette consisting of the *NOPALINE SYNTHASE* (*NOS*) promoter, *NEOMYCIN PHOSPHOTRANSFERASE II* (*NPTII*; kanamycin resistance) coding sequence, and NOS transcriptional terminator (Figure S1b). The multiple cloning site from the pPZP-RCS1 vector (Goderis et al., 2002) was then inserted between the selection cassette and the right T-DNA border. The amplified *MsWOX13-2* RNAi fragments were subsequently introduced into the pAUX3132 vector (Goderis et al., 2002) between a partially duplicated CaMV 35S promoter and intronic spacer from the pHannibal vector (Wesley et al., 2001) in the sense orientation, and between the intronic spacer and *NOS* terminator in the antisense orientation. The resulting RNAi cassette was then inserted into the binary background vector to generate the *MsWOX13-2* RNAi vector (Figure S1c), and was sequenced to confirm its identity. The empty vector control simply consisted of the background RNAi vector lacking the pAUX3132-derived RNAi cassette.

To generate the *MsWOX13-2* CRISPR vector, a guide RNA (gRNA; 20-nt) was designed immediately upstream of a 5′ - NGG - 3′ PAM sequence (Doench et al., 2014) within the homeodomain-encoding second exon of all 4 *MsW0X13-2* alleles (Figure S8a). The specificity and/or potential for off-target effects of the gRNA was confirmed using the Cas-OFFinder algorithm (Bae et al., 2014). Oligonucleotides corresponding to the gRNA in both orientations (Table S1) were synthesized by a service provider (Integrated DNA Technologies [IDT] Inc., Coralville, IA) and were inserted separately into the pKSE401 vector (Xing et al., 2014) as described previously (Singer et al., 2022). The resulting vector (*MsWOX13-2* CRISPR; Figure S8b) was sequenced to confirm its identity.

*Evaluation of nodulation and nitrogen fixation capacity*

*Sinorhizobium meliloti* strain Sm1021 was grown in 5 mL liquid tryptone/yeast extract (TY; Beringer, 1974) media overnight at 28°C, with shaking at 220 rpm. Subsequently, 1 mL of the

starter culture was transferred to 250 mL of liquid TY media, and incubated at 28°C until it reached an OD600 of approximately 1.0. Prior to inoculation, all equipment was surface sterilized using a 1% bleach solution, while water and vermiculite were sterilized by autoclaving. Ten uniformly rooted stem cuttings, which were grown in wet Oasis® Horticubes® (Oasis Growers Solutions, Kent, OH, USA) for 14 days, were selected for each genotype and transplanted into pots (6.6 cm × 6.6 cm × 8.7cm) containing vermiculite saturated with sterilized water. Each pot was placed inside a transparent polyethylene plastic bag with the top left open, and were then placed on pot holders inside a tray to prevent cross-contamination. The stem cuttings were then inoculated by applying 5 mL of the bacterial culture or sterilized water as a control. Pots were covered with a transparent lid under greenhouse conditions, and each pot was watered from the top with 10 mL distilled water every three days. Twenty-four days following inoculation, roots were examined and nodules were counted under a Luxo KFM 17253 magnifier (Luxo Lamp Corp., Port Chester, NY) and photographed with a SZX10 stereomicroscope (Olympus Corporation, Tokyo, Japan).

A separate estimation of nitrogen fixation capacity was conducted whereby plants were

grown in potting mixture with nitrogen fertilizer (Cornell soilless potting mix) and without

nitrogen fertilizer (Cornell soilless potting mix without nitrogen fertilizer). Rooted stem cuttings

were transplanted into pots measuring 6.6 cm × 6.6 cm × 8.7 cm, and 14 days after

transplantation, each pot was placed within a transparent polyethylene plastic bag, leaving the

top open, and inoculated with 25 mL of diluted bacterial culture as described above (OD600=1.30; diluted with water at a ratio of 1:10) or tap water as un-inoculated controls. The plants were then grown under greenhouse conditions for three weeks.

*Assessment of root aerenchyma production*

Root aerenchyma formation in alfalfa was assessed under control growth conditions, as well as following 14 and 28 days of waterlogging, when waterlogging symptoms were apparent in wild-type plants. Roots were washed with tap water and sections of primary roots located 5 to 10 cm below soil level were harvested. Subsequently, root cross sections were manually prepared with a sharp razor blade, fixed with Image-iT™ Fixative Solutions (Thermo Fisher Scientific) for 15 minutes, and stained with 0.025% (w/v) toluidine blue (Spectrum, New Brunswick, USA) for 5 minutes. The sections were then mounted on microscope slides, covered with coverslips, dried for 1 to 2 minutes at 60°C, and observed and photographed using an SZX10 stereomicroscope under 6×magnification.

*RNA-Seq analysis*

RNA integrity was assessed using the RNA Nano 6000 Assay Kit and a Bioanalyzer 2100 system (Agilent Technologies, Santa Clara, CA, USA), and stranded mRNA library preparation was carried out using 1 µg total RNA and the NEBNext® Ultra™ RNA Library Prep kit (New

England Biolabs, Whitby, ON). Subsequently, sequencing was carried out on an Illumina NovaSeq 6000 platform (Illumina Inc., San Diego, CA), generating 150-bp paired-end reads. Raw data were cleaned and trimmed using in-house perl scripts (Novogene, Sacramento, California, USA), and the resulting data were then mapped to the *Medicago truncatula* reference genome (v. 4.01.4), which is a close diploid relative of alfalfa with a very well-annotated genome, using Hisat2 v2.0.5 (Kim et al., 2015).

Gene expression levels were determined using featureCounts v1.5.0-p3 (Liao et al., 2014), which allowed the calculation of fragments per kilobase of transcript per million mapped reads (FPKM) for each gene. Differential expression analysis was performed using the DESeq2 R package (1.20.0) (Love et al., 2014). The Benjamini and Hochberg's approach was used to adjust p-values for controlling false discovery rate, and genes with a p-value < 0.05, were considered differentially expressed genes (DEGs). Principal component analysis (PCA) and cluster analysis (heat maps) were generated using the R packages ggplot2 and pheatmap, respectively. Gene ontology (GO) term enrichment analysis was conducted using the clusterProfiler R package (Yu et al., 2012). GO terms with a corrected p-value < 0.05 were considered significantly enriched.

Weighted gene co-expression network analysis (WGCNA) was carried out using the WGCNA R package (Langfelder & Horvath, 2008). Gene-level count data from DESeq2-processed RNA-Seq libraries across all samples (two RNAi genotypes and a wild-type genotype, under normally-watered and waterlogged conditions) were first normalized using variance stabilizing transformation (VST). A signed, scale-free network was constructed using a soft-thresholding power of 10, chosen based on scale-free topology fit and mean connectivity criteria. Modules were detected using hierarchical clustering with dynamic tree cut (minimum module size = 30), and similar modules were merged using a cut height of 0.2. Each module was summarized by its eigengene (first principal component), and module–trait associations were computed using Pearson correlation between eigengenes and sample traits (genotype and growth condition). Functional enrichment for selected modules was conducted using GO terms with GOATOOLS (Klopfenstein et al., 2018). The top 30 hub genes in each module were determined using the WGCNA softConnectivity function.

**References**

**Bae, S., Park, J., & Kim, J.S.** (2014) Cas-OFFinder: a fast and versatile algorithm that searches for potential off-target sites of Cas9 RNA-guided endonucleases**.** *Bioinformatics*, **30**, 1473-1475.

**Chen, H., Zeng, Y., Yang, Y., Huang, L., Tang, B., Zhang, H. *et al.*** (2020) Allele-aware chromosome-level genome assembly and efficient transgene-free genome editing for the autotetraploid cultivated alfalfa. *Nature Communications*, **11**, 2494.

**Goderis, I.J., De Bolle, M.F., François, I.E., Wouters, P.F., Broekaert, W.F., Cammue, B.P. *et al.*** (2002) A set of modular plant transformation vectors allowing flexible insertion of up to six expression units. *Plant Molecular Biology,* **50**, 17-27.

**Kim, D., Langmead, B.** & **Salzberg, S.L.** (2015) HISAT: a fast spliced aligner with low memory requirements. *Nature Methods*, **12**, 357-360.

**Langfelder, P. & Horvath, S.** (2008) WGCNA: an R package for weighted correlation network analysis. *BMC bioinformatics*, **9**, 1-13.

**Lia, Y., Smyth, G.K. & Shi, W.** (2014) featureCounts: an efficient general purpose program for assigning sequence reads to genomic features. *Bioinformatics*, **30**, 923-930.

**Klopfenstein, D.V., Zhang, L., Pedersen B.S., Ramírez, F., Vesztrocy, A.W., Naldi, A., *et al*.** (2018) GOATOOLS: A python library for gene ontology analyses. *Scientific Reports*, **8**, 10872.

**Love, M.I., Huber, W.** & **Anders, S.** (2014) Moderated estimation of fold change and dispersion for RNA-seq data with DESeq2. *Genome Biology,* **15**, 1-21*.*

**Mann, D.G.J., LaFayette, P.R., Abercrombie, L.L., King, Z.R., Mazarei, M., Halter, M.C., *et al.*** (2012) Gateway-compatible vectors for high-throughput gene functional analysis in switchgrass (*Panicum virgatum* L.) and other monocot species. *Plant Biotechnology Journal*, **10**, 226-236.

**Singer, S.D., Burton Hughes, K., Subedi, U., Dhariwal, G.K., Kader, K., Acharya, S. *et al.*** (2022) The CRISPR/Cas9-mediated modulation of *SQUAMOSA PROMOTER-BINDING PROTEIN-LIKE 8* in alfalfa leads to distinct phenotypic outcomes. *Frontiers in Plant Science*, **12**, 774146.

**Wesley, S. V., Helliwell, C. A., Smith, N. A., Wang, M., Rouse, D. T., Liu, Q. *et al.*** (2001) Construct design for efficient, effective and high‐throughput gene silencing in plants. *The Plant Journal*, **27**, 581-590.

**Xing, H. L., Dong, L., Wang, Z. P., Zhang, H. Y., Han, C. Y., Liu, B. *et al.*** (2014) A CRISPR/Cas9 toolkit for multiplex genome editing in plants. *BMC plant biology*, **14**, 1-12.

**Yu, G.**, **Wang, L.G.**, **Han, Y.** & **He, Q.Y.** (2012) clusterProfiler: an R package for comparing biological themes among gene clusters. *Omics*, **16**, 284-287.
