# Supplementary material for: The down‐regulation of MsWOX13‐2 promotes enhanced waterlogging resilience in alfalfa
Source: Plant J. 2025 Aug 20;123(4):e70411. doi: 10.1111/tpj.70411 (PMC12368323; doi:10.1111/tpj.70411)
Supplement: Supplementary file 6 — Figure S1. Gene structure, vectors, and assessment of MsWOX13‐2 and MsWOX13‐1 transcript levels in MsWOX13‐2 RNAi genotypes. Figure S2. Impact of MsWOX13‐2 down‐regulation on nodulation in alfalfa. Figure S3. Root cross‐sections for the visualization of aerenchyma in MsWOX13‐2 RNAi and wild‐type genotypes under normally watered conditions and after 14 and 28 days of waterlogging. Figure S4. Analysis of differentially expressed genes (DEGs) in wild‐type and MsWOX13‐2 RNAi leaf tissues under normally watered conditions and after waterlogging for 14 days. Figure S5. Gene Ontology (GO) term enrichment analysis of differentially expressed genes (DEGs) between wild‐type and MsWOX13‐2 RNAi genotypes under normally watered (a) and waterlogged (b) conditions. Figure S6. Transcriptional alteration of genes involved in abiotic stress‐related pathways, redox, transcription factor families, and phytohormonal regulation in MsWOX13‐2 RNAi genotypes compared to wild‐type under waterlogging stress. Figure S7. Weighted gene co‐expression network analysis (WGCNA) of RNA‐Seq data from MsWOX13‐2 RNAi and wild‐type genotypes under normally watered and waterlogged conditions. Figure S8. CRISPR/Cas9‐mediated gene editing of MsWOX13‐2 in alfalfa. Figure S9. Identification of MsWOX13‐2 edits and confirmation of a lack of off‐target mutations. Figure S10. Waterlogging resilience of MsWOX13‐2 CRISPR genotypes compared to empty vector genotypes. [file TPJ-123-0-s004.pdf]

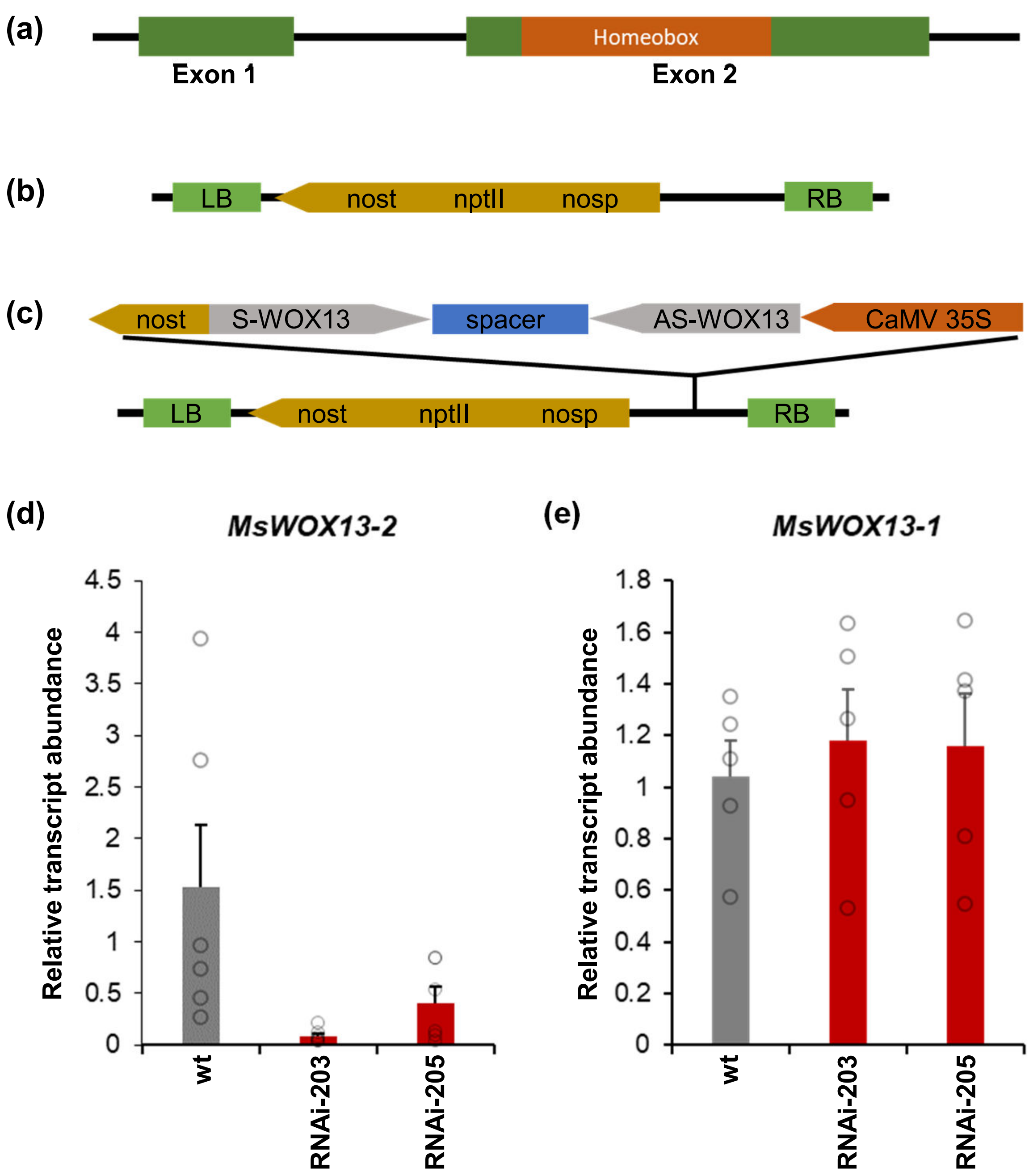

Figure S1. Gene structure, vectors, and assessment of *MsWOX13-2* and *MsWOX13-1* transcript levels in *MsWOX13-2* RNAi genotypes. (**a**) Schematic diagram (not to scale) of *MsWOX13-2*. (**b**) Schematic diagram (not to scale) of the empty vector and (**c**) *MsWOX13-2* RNAi vector. (**d**) Expression level of *MsWOX13-2* in wild-type and *MsWOX13-2* RNAi genotypes. (**e**) Expression levels of *MsWOX13-1* in wild-type and *MsWOX13-2* RNAi genotypes. Blocks represent the means of 5 to 6 biological replicates of each genotype and bars indicate standard errors. S-WOX13, sense *MsWOX13-2* RNAi fragment; AS-WOX13, antisense *MsWOX13-2* RNAi fragment, LB, left border; nosp, nos promoter; nost, nos terminator; nptII; neomycin phosphotransferase II; RB, right border; wt, N4.4.2 wild-type alfalfa genotype.

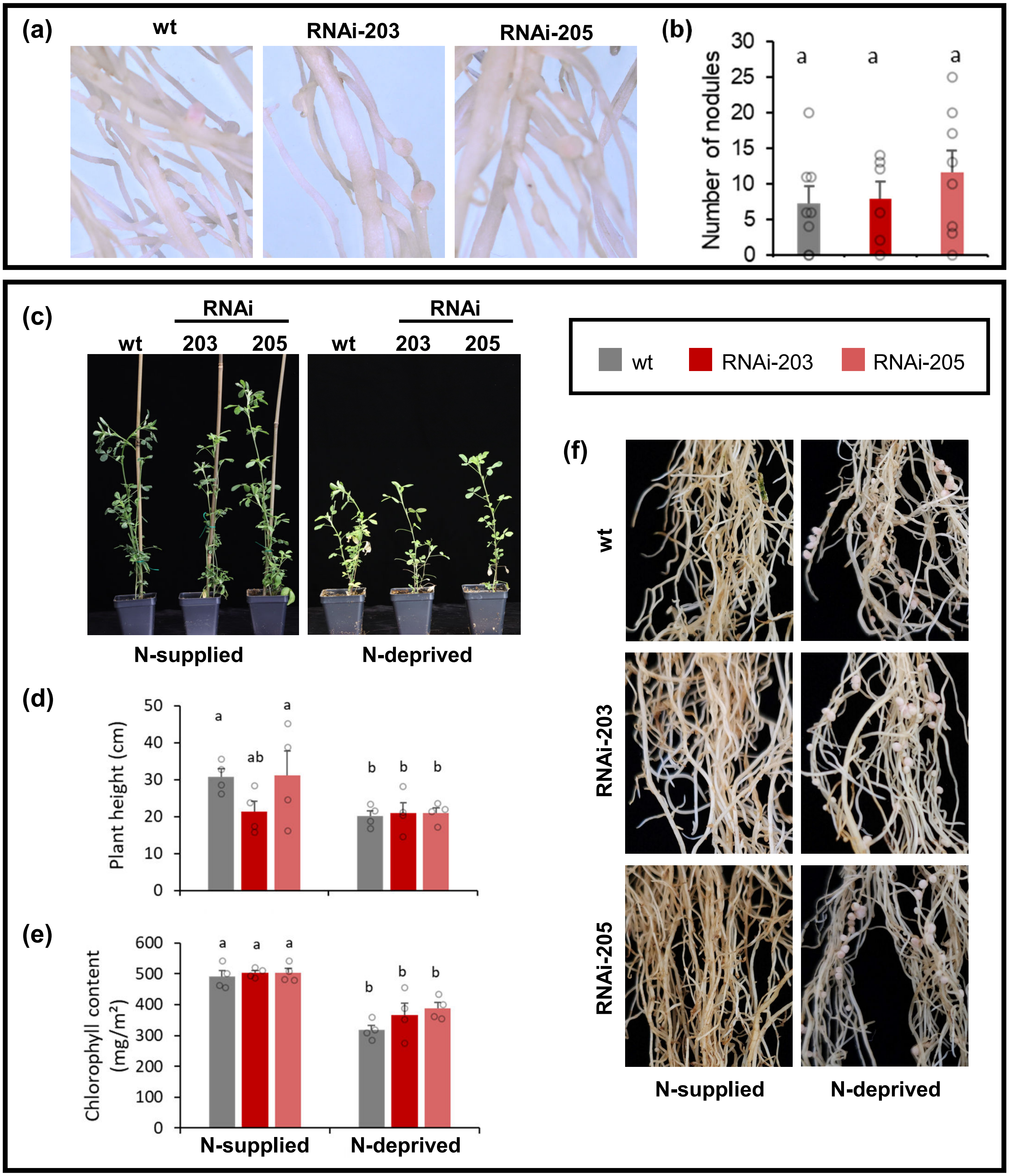

Figure S2. Impact of *MsWOX13-2* down-regulation on nodulation in alfalfa. **(a)** Representative images of roots with nodules from wild-type and *MsWOX13-2* RNAi genotypes 24 days after inoculation with *Sinorhizobium meliloti*. **(b)** Total number of nodules 24 days after rhizobial inoculation. **(c)** Representative images of plants, **(d)** plant height, **(e)** chlorophyll content, and **(f)** representative images of roots with nodules from wild-type and *MsWOX13-2* RNAi genotypes 21 days after rhizobial inoculation in nitrogen-supplied and -deprived soil. Blocks represent the means of 6 to 8 **(b)** or 4 **(d, e)** biological replicates of each genotype, and bars denote standard errors. Lower case letters indicate statistically significant differences between groups as determined by one-way **(b)** or two-way **(d, e)** ANOVA followed by Tukey's HSD test ($p \leq 0.05$).

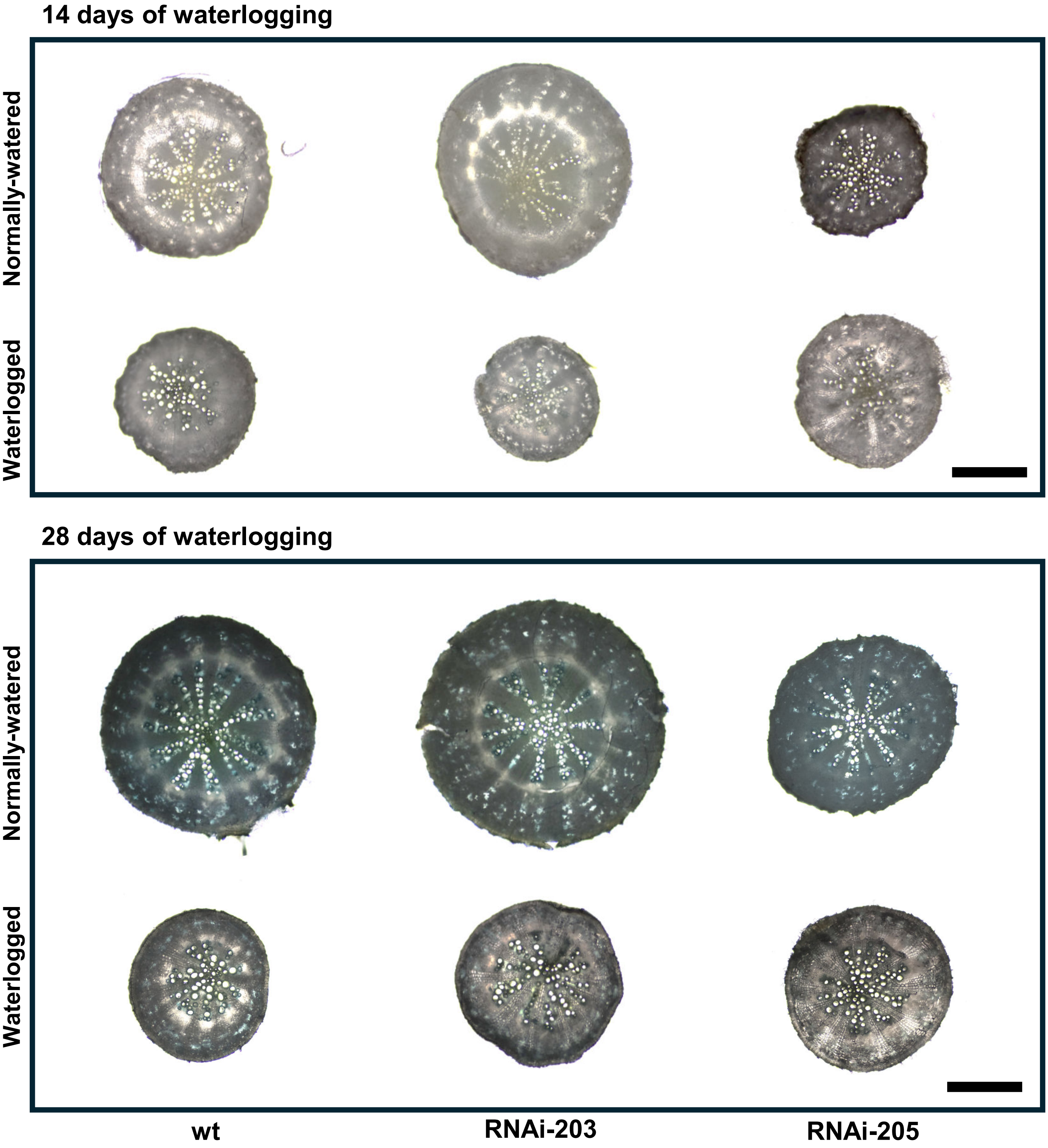

Figure S3. Root cross-sections for the visualization of aerenchyma in *MsWOX13-2* RNAi and wild-type genotypes under normally-watered conditions and after 14 and 28 days of waterlogging. Root cross-sections were prepared from 3 biological replicates of each genotype, and from each biological replicate, two distinct root sections were taken from a region 5-10 cm below the soil surface. Scale bars indicate a length of 640 μm.

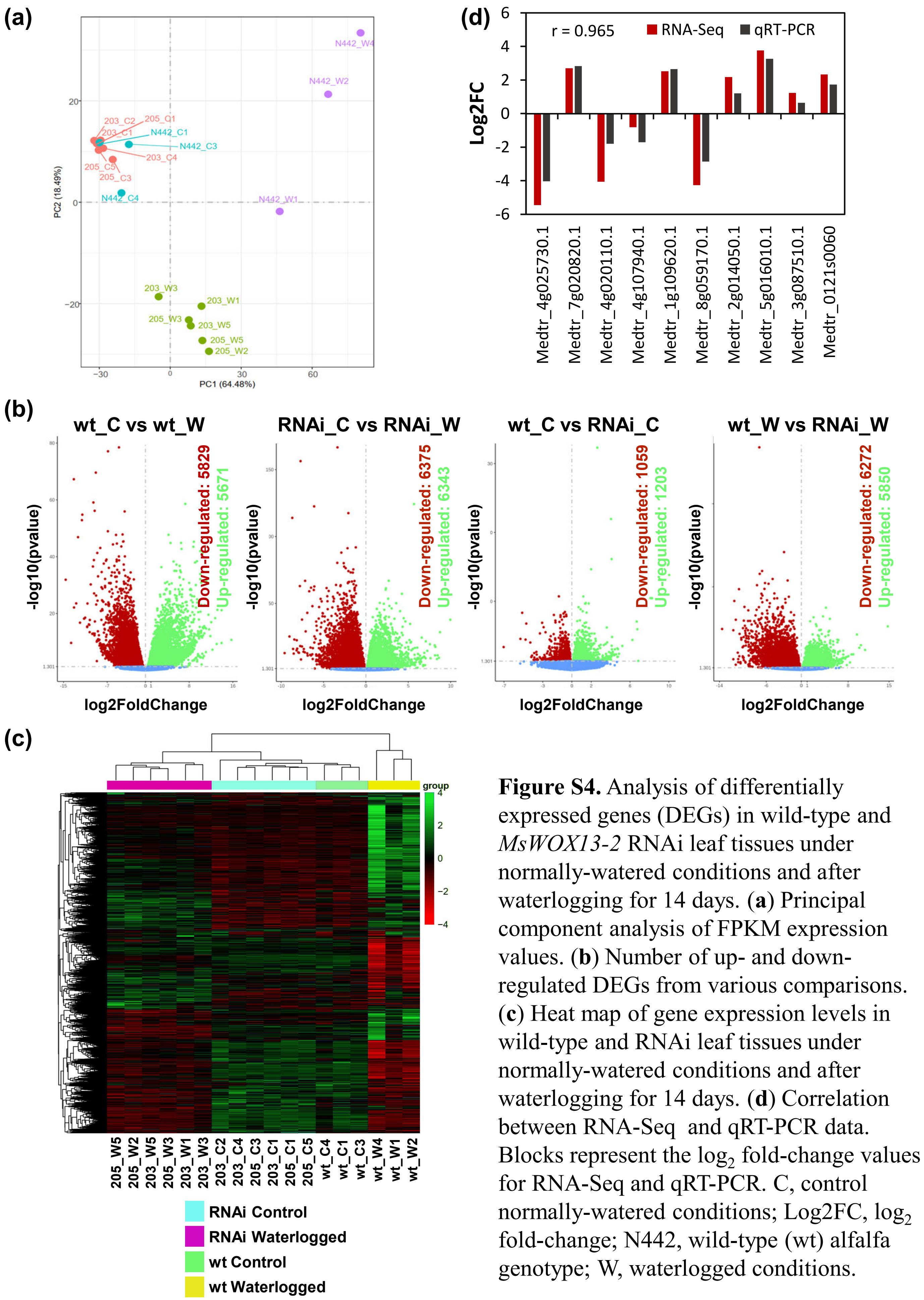

Figure S4. Analysis of differentially expressed genes (DEGs) in wild-type and *MsWOX13-2* RNAi leaf tissues under normally-watered conditions and after waterlogging for 14 days. (**a**) Principal component analysis of FPKM expression values. (**b**) Number of up- and down-regulated DEGs from various comparisons. (**c**) Heat map of gene expression levels in wild-type and RNAi leaf tissues under normally-watered conditions and after waterlogging for 14 days. (**d**) Correlation between RNA-Seq and qRT-PCR data. Blocks represent the log_2 fold-change values for RNA-Seq and qRT-PCR. C, control normally-watered conditions; Log2FC, log_2 fold-change; N442, wild-type (wt) alfalfa genotype; W, waterlogged conditions.

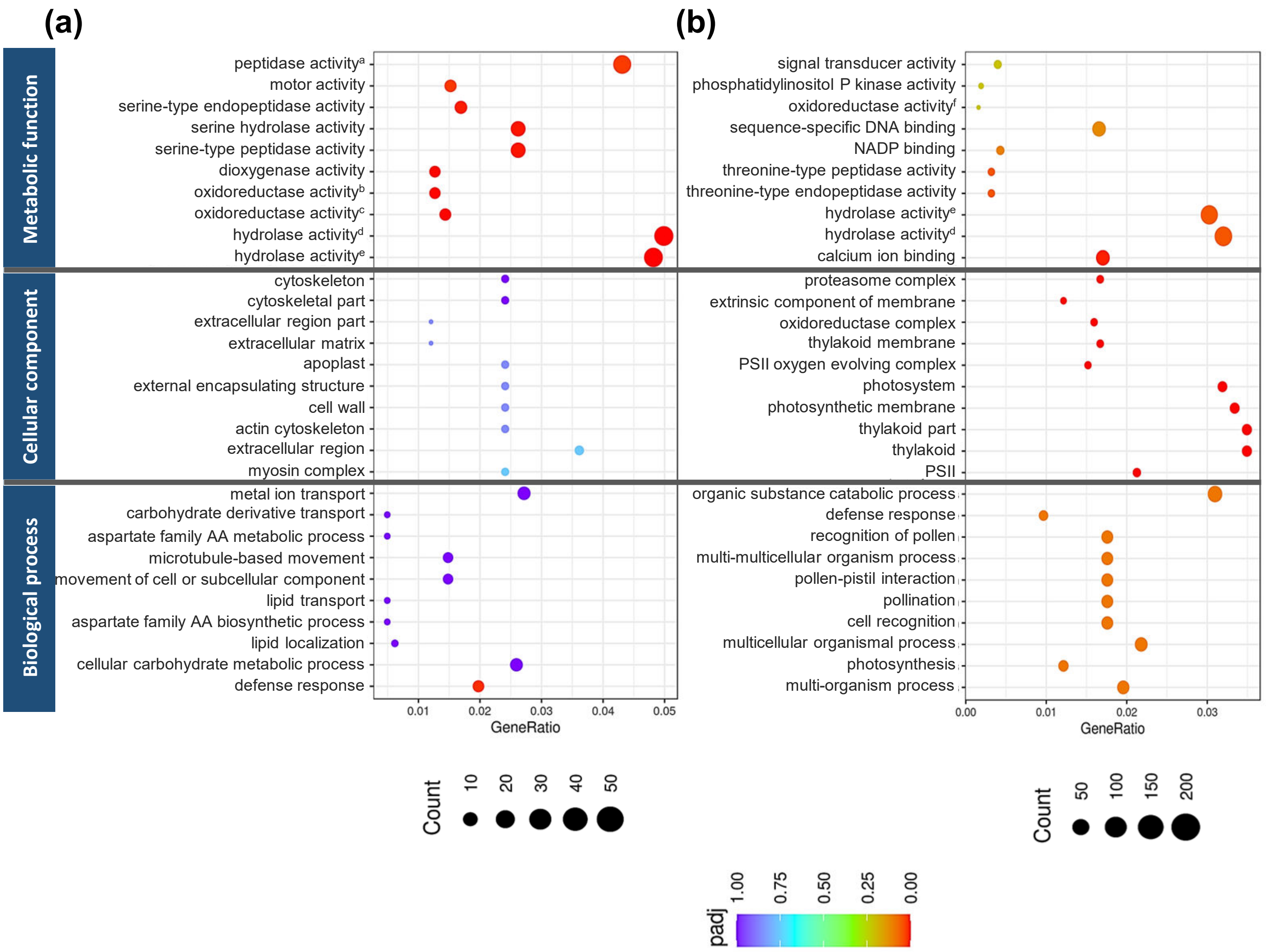

Figure S5. Gene Ontology (GO) term enrichment analysis of differentially expressed genes (DEGs) between wild-type and *MsWOX13-2* RNAi genotypes under normally-watered (**a**) and waterlogged (**b**) conditions. The y-axis displays GO terms, while the x-axis represents the ratio of DEGs to the number of annotated genes in each GO term. Dot size indicates the number of DEGs enriched in a particular GO term. The color of the dot represents the adjusted *p*-value. Peptidase activity[a] = peptidase activity, acting on L-amino acid peptides; Oxidoreductase activity[b] = oxidoreductase activity acting on single donors with incorporation of molecular oxygen, incorporation of two atoms of oxygen; oxidoreductase activity[c] = oxidoreductase activity acting on single donors with incorporation of molecular oxygen; hydrolase activity[d] = hydrolase activity, acting on glycosyl bonds; hydrolase activity[e] = hydrolase activity, hydrolyzing O-glycosyl compounds; oxidoreductase activity[i] = oxidoreductase activity, acting on diphenols and related substances as donors. AA, amino acid; P, phosphate; PSII, photosystem II.

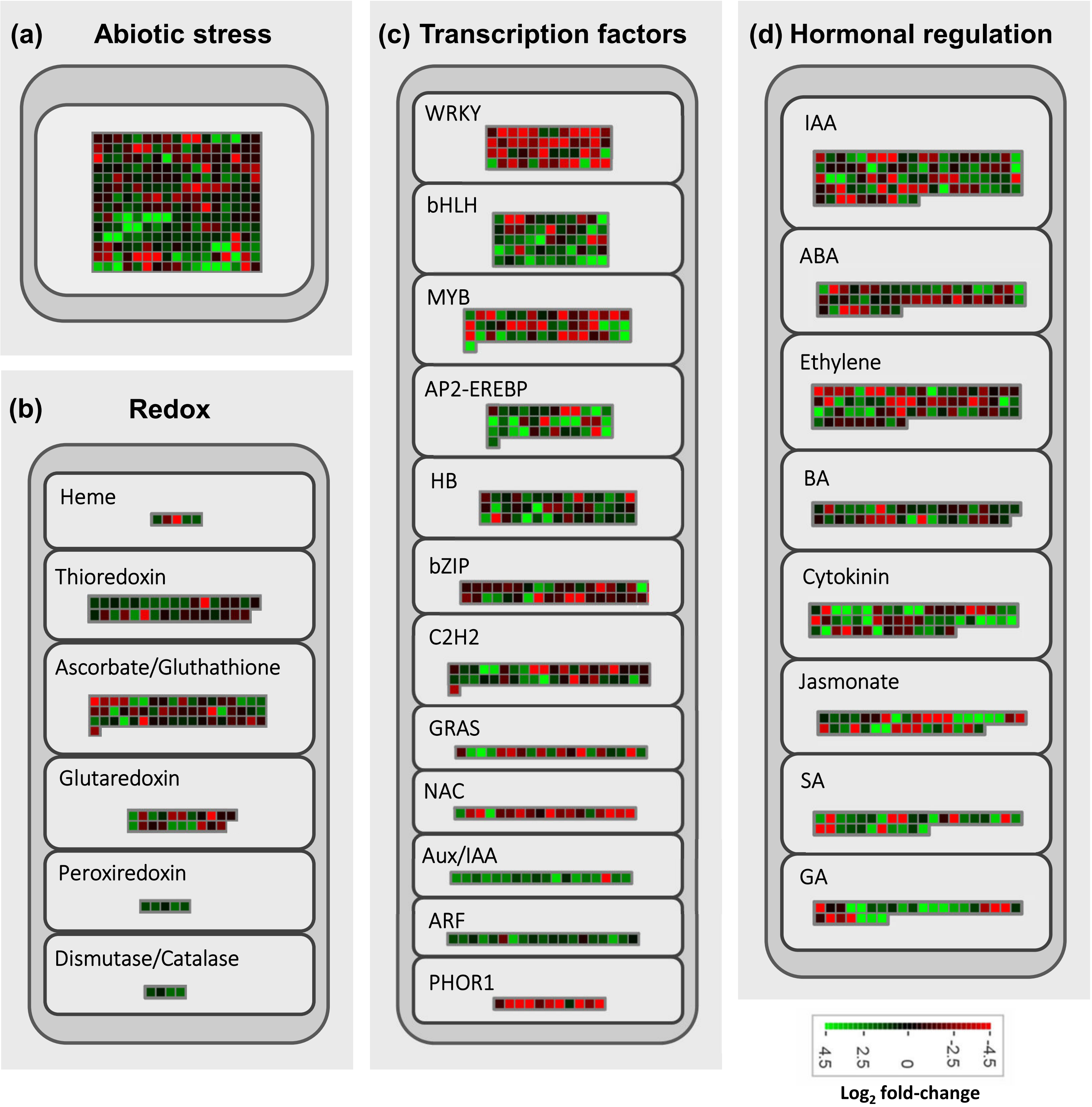

Figure S6. Transcriptional alteration of genes involved in abiotic stress-related pathways, redox, transcription factor families, and hormonal regulation in *MsWOX13-2* RNAi genotypes compared to wild-type under waterlogging stress. Differentially expressed genes involved in (**a**) abiotic stress response, (**b**) redox, (**c**) transcription factors, and (**d**) hormonal regulation. Pathway analysis was conducted using MapMan, with green boxes indicating up-regulated genes and red boxes denoting down-regulated genes.

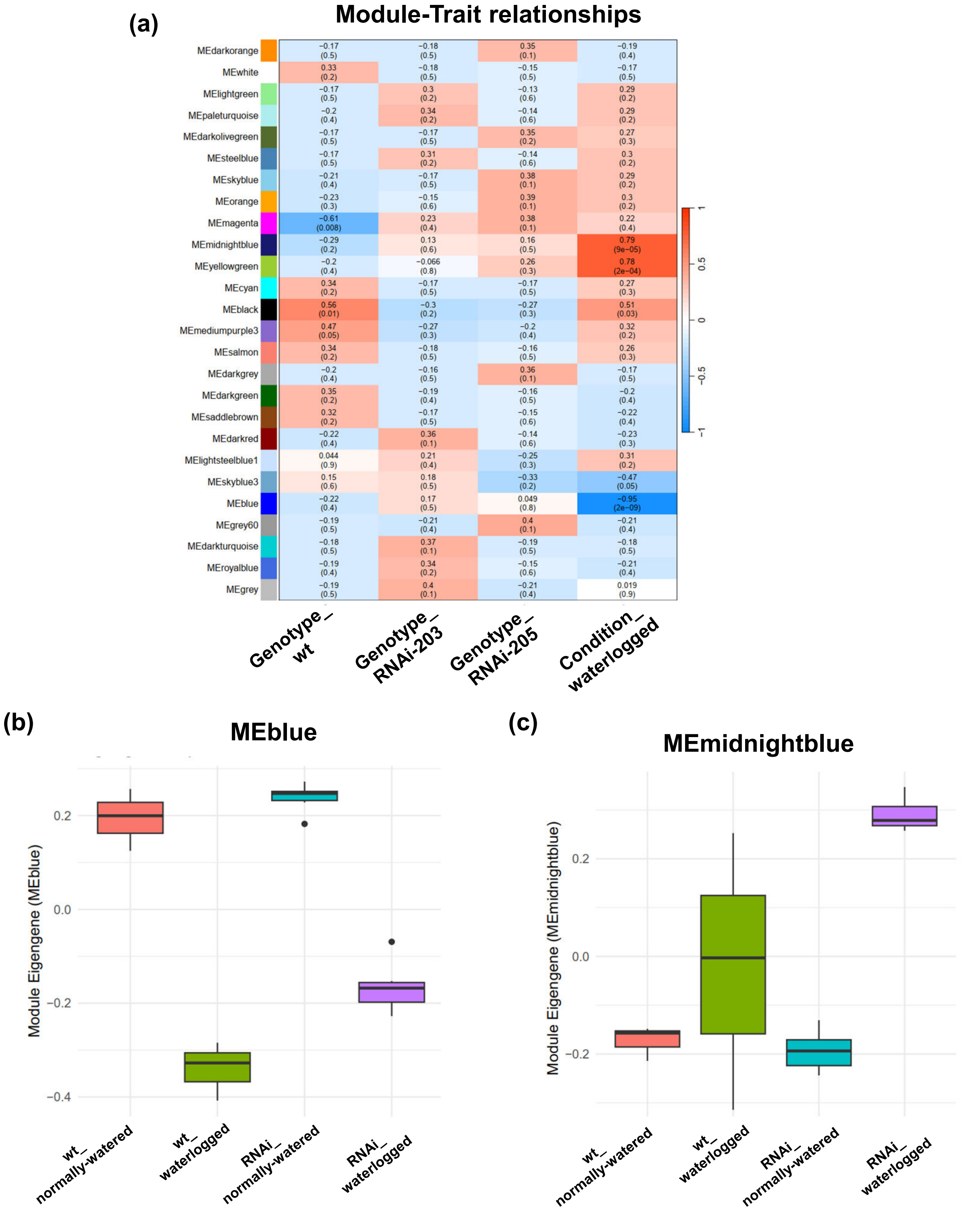

Figure S7. Weighted gene co-expression network analysis (WGCNA) of RNA-Seq data from *MsWOX13-2* RNAi and wild-type genotypes under normally-watered and waterlogged conditions. **(a)** Module-trait correlation heatmap showing the association between module eigengenes and genotype or treatment conditions. Each cell indicates the Pearson correlation coefficient (top) and corresponding p-value (bottom). **(b)** Boxplot of module eigengene (ME) expression for the "blue" module. **(c)** Boxplot of eigengene expression for the "midnightblue" module.

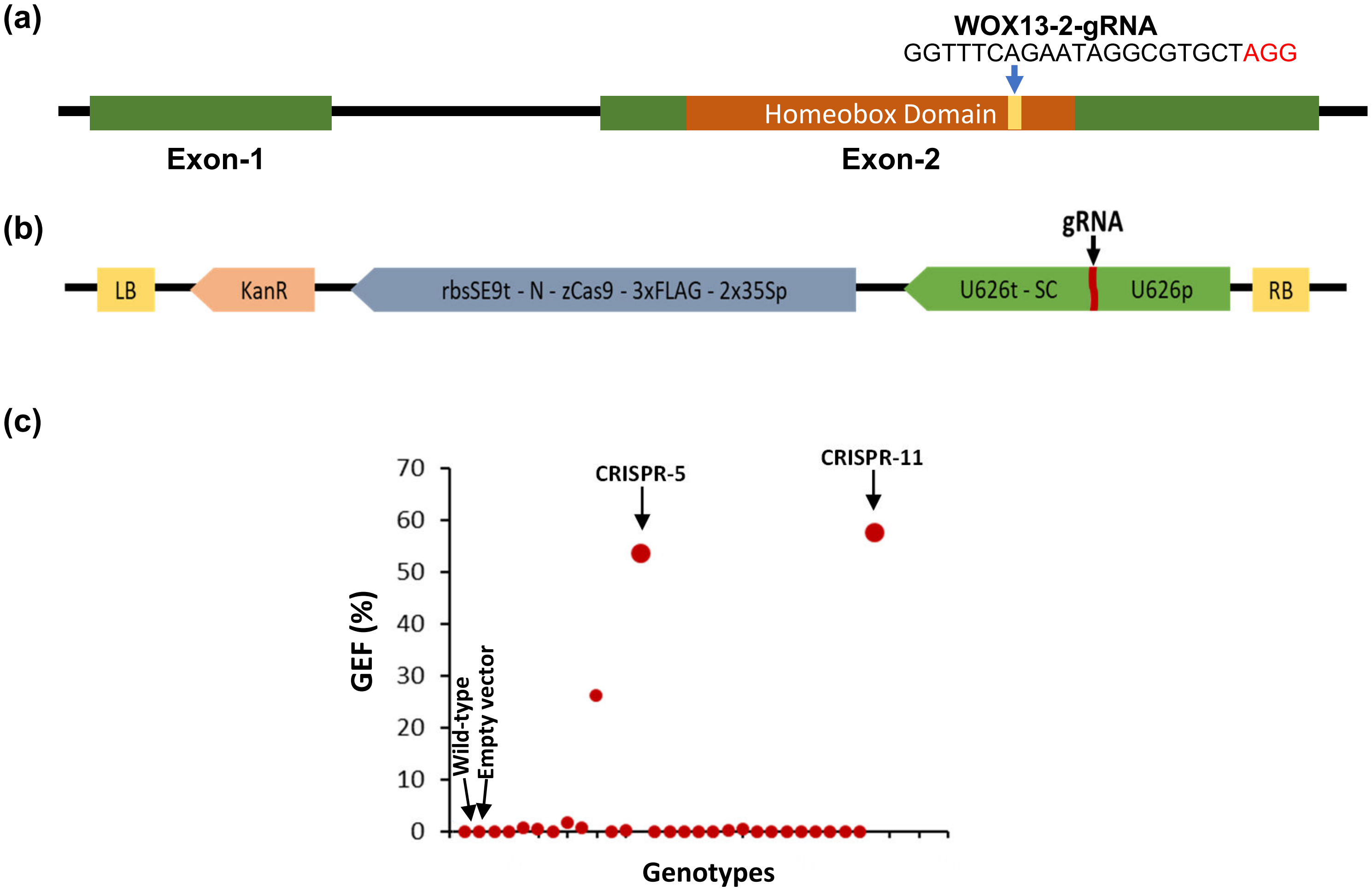

Figure S8. CRISPR/Cas9-mediated gene editing of *MsWOX13-2* in alfalfa. **(a)** Schematic diagram (not to scale) of the target site for the *MsWOX13-2*-specific gRNA. The PAM sequence associated with the gRNA is indicated in red. **(b)** Schematic diagram (not to scale) of the editing vector. **(c)** Gene editing frequencies (GEFs) of each transgenic *WOX13-2* CRISPR genotype (red dots). 2x35Sp, partially duplicated CaMV 35S promoter; 3xFLAG, polypeptide tag; KanR, kanamycin resistance cassette; LB, left border; N, nuclear localization signal; RB, right border; rbcSE9t, pea RuBisCO small subunit E9 terminator; SC, sgRNA scaffold; U626p; Arabidopsis U6 polymerase III promoter; U626t; U6 terminator; zCas9, *Zea mays* codon-optimized Cas9.

(a)

	TAATTGGTTTCAGAAT------GCTAGGTCAAAGGGAAAGCAGCAAA	**6 bp del**
**CRISPR-5**	TAATTGGTTTCAGAATAGGCGTT-----------------------A	**24 bp del, 1 bp subs**
	TAATTGGTTTCAGAATAGGCGTGCTAGGTCAAAGGGAAAGCAGCAAA	**wild-type**
	TAATTGGTTTCAGAATAGG----------------GAAAGCAGCAAA	**16 bp del**
**CRISPR-11**	TAA------------------------------GGGAAAGCAGCAAA	**30 bp del**
	TAATTGGTTTCAGAATAGGCGTGCTAGGTCAAAGGGAAAGCAGCAAA	**wild-type**

(b)

	EKIKEITADLTKHGPISETSVYNWFQN--ARSKGKQQNN...	**2 aa del**
**CRISPR-5**	EKIKEITADLTKHGPISETSVYNWFQNRR*	**truncation**
	EKIKEITADLTKHGPISETSVYNWFQNRRARSKGKQQNN…	**wild-type**
	EKIKEITADLTKHGPISETSVYNWFQNRESSKIMLMMNRK*	**truncation**
**CRISPR-11**	EKIKEITADLTKHGPISETSVY----------GKAAK*	**truncation**
	EKIKEITADLTKHGPISETSVYNWFQNRRARSKGKQQNN…	**wild-type**

Figure S9. Identification of *MsWOX13-2* edits and confirmation of a lack of off-target mutations. **(a)** Nucleotide sequences of *MsWOX13-2* alleles encompassing the WOX13-2-gRNA target region from 2 selected CRISPR/Cas9-edited genotypes with gene editing frequences of approximately 50%. Green highlighting indicates a nucleotide substitution, while dashed lines indicate deleted nucleotides. **(b)** Corresponding amino acid sequences of MsWOX13-2 from the WOX13-2-gRNA target region from the 2 selected CRISPR/Cas9-edited genotypes. Asterisks denote the presence of a stop codon. aa, amino acid; del, deletion; subs, substitution.

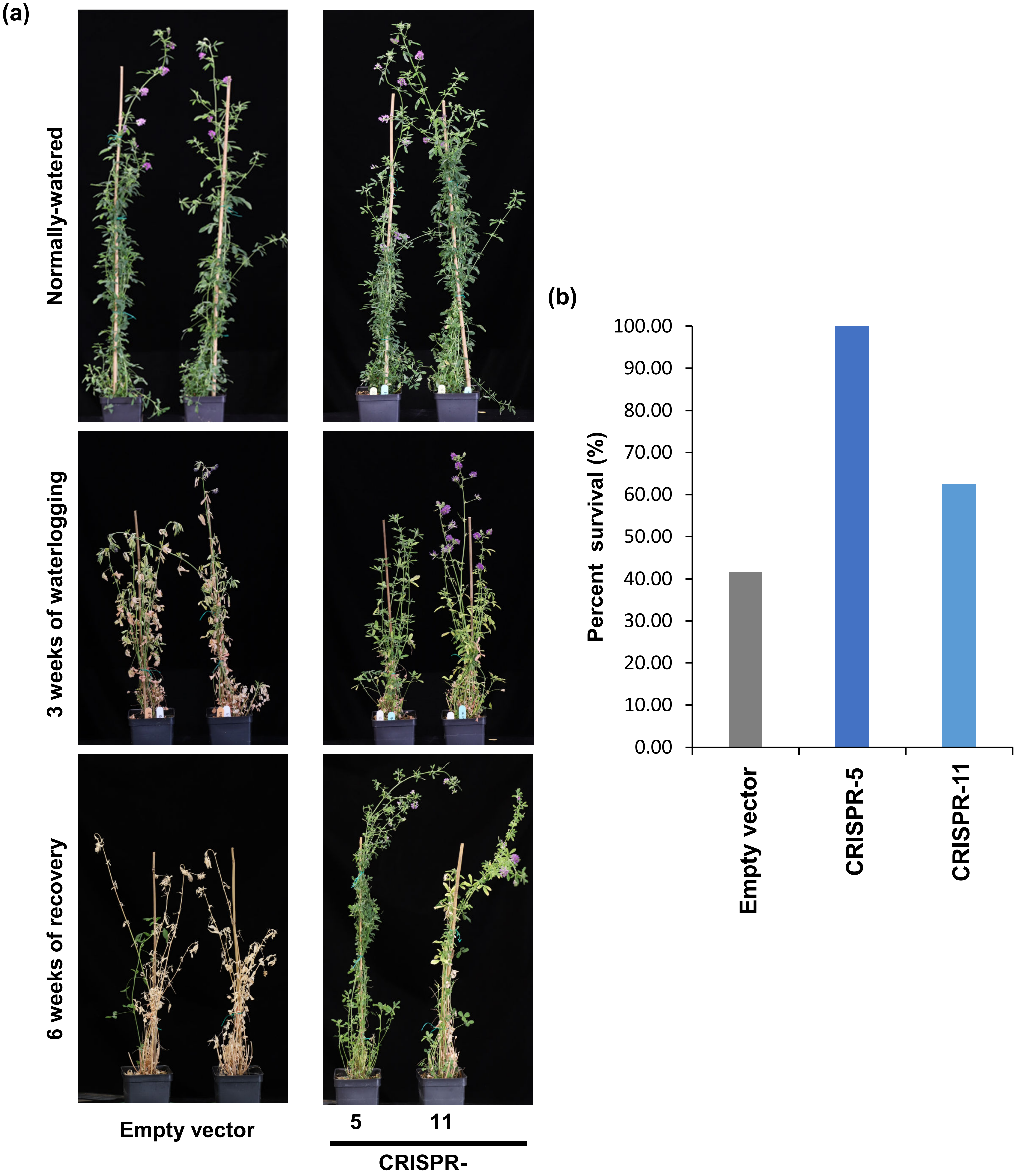

Figure S10. Waterlogging resilience of *MsWOX13-2* CRISPR genotypes compared to empty vector genotypes. **(a)** Representative images of *MsWOX13-2* CRISPR (CRISPR-5 and CRISPR-11) and empty vector genotypes grown under normally-watered conditions, after 3 weeks of waterlogging, and after 6 weeks of recovery. **(b)** Percent survival of plants following 6 weeks of recovery after 3 weeks of waterlogging treatment. Blocks represent the means of 8 biological replicates from each *MsWOX13-2* CRISPR genotype and 12 biological replicates from three independent empty vector genotypes (pooled).
