# Supplementary material for: The down‐regulation of MsWOX13‐2 promotes enhanced waterlogging resilience in alfalfa
Source: Plant J. 2025 Aug 20;123(4):e70411. doi: 10.1111/tpj.70411 (PMC12368323; doi:10.1111/tpj.70411)
Supplement: Supplementary file 7 — Table S1. Summary of Illumina sequencing data and mapped reads for each sample. Table S2. Off‐target mutation analysis. Table S3. Primers and probes used in vector construction, qRT‐PCR assessments of MsWOX13 transcript levels, and the analysis of edited genotypes. Table S4. List of primers used for the qRT‐PCR validation of RNA‐Seq results. [file TPJ-123-0-s003.docx]

**Table S1:** Summary of Illumina sequencing data and mapped reads for each sample.

| **Sample** | **Clean reads** | **Clean bases** | **GC %** | **Total mapped reads** |
| --- | --- | --- | --- | --- |
| **N442_C1** | 1.03E+08 | 15.4G | 43.12 | 635,26,539 (61.88%) |
| **N442_C3** | 1.32E+08 | 19.87G | 43.15 | 82,269,654 (62.11%) |
| **N442_C4** | 1.12E+08 | 16.77G | 42.7 | 68,240,420 (61.03%) |
| **N442_W1** | 1.44E+08 | 21.64G | 41.6 | 79,475,323 (55.08%) |
| **N442_W2** | 1.33E+08 | 19.96G | 41.43 | 70,794,211 (53.2%) |
| **N442_W4** | 1.04E+08 | 15.64G | 41.29 | 55,336,457 (53.06%) |
| **WOX13_203_C1** | 1.09E+08 | 16.39G | 42.98 | 66,184,586 (60.56%) |
| **WOX13_203_C2** | 1.06E+08 | 15.86G | 43.07 | 64,281,452 (60.8%) |
| **WOX13_203_C4** | 1.01E+08 | 15.11G | 43.01 | 60,765,898 (60.31%) |
| **WOX13_203_W1** | 1.01E+08 | 15.12G | 42.21 | 57,235,547 (56.79%) |
| **WOX13_203_W3** | 1.52E+08 | 22.79G | 42.34 | 87,736,216 (57.74%) |
| **WOX13_203_W5** | 1.35E+08 | 20.27G | 42.14 | 76,941,656 (56.93%) |
| **WOX13_205_C1** | 1.37E+08 | 20.58G | 43.1 | 84,506,135 (61.6%) |
| **WOX13_205_C3** | 1.59E+08 | 23.79G | 43.1 | 97,201,213 (61.27%) |
| **WOX13_205_C5** | 1.09E+08 | 16.37G | 43.15 | 66,895,357 (61.3%) |
| **WOX13_205_W2** | 1.47E+08 | 22.11G | 41.92 | 82,414,378 (55.9%) |
| **WOX13_205_W3** | 1.62E+08 | 24.3G | 41.99 | 93,146,404 (57.5%) |
| **WOX13_205_W5** | 1.2E+08 | 17.97G | 42.15 | 69,017,214 (57.6%) |

C1-C5, biological replicates grown under control conditions; W1-W5, biological replicates grown under waterlogged conditions; N442, wild-type N4.4.2 genotype; WOX13_203, RNAi-203 genotype; WOX13_205, RNAi-205 genotype.

**Table S2:** Off-target mutation analysis.

| **Genotype** | **Off-target site** | **Putative off-target sequences**  **(20 nt gRNA and 3 nt PAM)** | **Number of clones examined** | **Number of clones with mutations** |
| --- | --- | --- | --- | --- |
| WOX13-2-gRNA2-5 | 1 | GGTTcCAaAATAGGCGTGCTAGG | 5 | 0 |
| WOX13-2-gRNA2-11 | 1 | GGTTcCAaAATAGGCGTGCTAGG | 8 | 0 |

PAM sequences are denoted in red; nucleotides that differ from the gRNA sequence are indicated in lowercase.

| **Primer/probe names** | **Primer sequence (5’ – 3’; engineered restriction sites italicized)** | **Amplicon length (bp)** |
| --- | --- | --- |
| MsWOX13-2RNAiF1EcoRI  MsWOX13-2RNAiR1PstI  MsWOX13-2RNAiF1XhoI  MsWOX13-2RNAiR1KpnI | *GAATTC*TAGGCTGAGGCAGCGTTGG  *CTGCAG*TCCGCTAAGTACTGGCTGCA  *CTCGAG*CTAGGCTGAGGCAGCGTTGG  *GGTACC*TCCGCTAAGTACTGGCTGCA | 319 |
| MsWOX13-1qPCRF1  MsWOX13-1qPCRR1 | GGGAATGTCGAAGCGATGTAT  CAAGCTGTTCACAAATGGTAGC | 100 |
| MsWOX13-2qPCRF1  MsWOX13-2qPCRR1 | AGTCGAAACTCTTCGGAAACA  AGACCCAGTGAGAGTGAGAT | 109 |
| MsADFF1  MsADFR1 | GCATCTGGTATGGCAGTCC  GCACTCATCAGCAGGAAGG | 183 |
| MsWOX13 gRNAddPCRF1 MsWOX13 gRNAddPCRR1 | ACCAACACCTGTGCAACT  CAGTTTTCTTATCATTGGAATCAACC | 239 |
| MsWOX13geneseqF1  MsWOX13geneseqR1 | GTGGAGCCCGGTTCAATATAA  AGAGCGTCGTAAACTGTCATC | 473 |
| MsWOX13-gRNA LNA probe | ATAGGCGTGCTAG | NA |
| MsWOX13-gRNA FAM probe | TGGAGCGAATATTTGAAGCTGAGACGG | NA |
| WOX13gRNAoffF1  WOX13gRNAoffR1 | AACGAGTGAACAGATGGAATCA  TCCTTGGACACAACCTCAAAT | 210 |

**Table S3:** Primers and probes used in vector construction, qRT-PCR assessments of *MsWOX13* transcript levels, and the analysis of edited genotypes.

**Table S4:** List of primers used for the qRT-PCR validation of RNA-Seq results.

| **Gene ID** | **Annotation** | **Primer sequences (5’ – 3’)** | **Amplicon length (bp)** |
| --- | --- | --- | --- |
| Medtr4g025730 | Pectinesterase/pectinesterase inhibitor | F: GCTGCTACTGGTGATGGATTTA  R: ATGCAACAGCTTGGTGATTTG | 82 |
| Medtr7g020820 | Proline dehydrogenase | F: GCTGAGATGGCAGAAGAAAGA  R: GCTCTGGTCTCTTCCTTGTATG | 110 |
| Medtr4g020110 | Delta1-pyrroline-5-carboxylate synthetase | F: ATGCTGGCATCCCTGTTATT  R: CGCGCTCTCTTCCTTTATGT | 134 |
| Medtr4g107940 | Delta1-pyrroline-5-carboxylate dehydrogenase | F: ACCAGAGCTTCAAGGGTTTC  R: TCTACTGTGGCAAGTGGTATTG | 98 |
| Medtr1g109620 | Trehalose-6-phosphate synthase domain protein | F: GGGTATTGTTGCAGAACGTATTC  R: GATAGAGATGCCCTTGCACTT | 135 |
| Medtr8g059170 | NAC transcription factor-like protein | F: GGGATGCTGAATTACGGTTGTA  R: GTCGGTGACCCGGTTAG | 138 |
| Medtr2g014050 | Late embryogenesis abundant domain protein | F: GAAGAATGCAGGAGATCAGGC  R: AATATCCCTTACTCCTTCCCAGC | 90 |
| Medtr5g016010 | Peroxidase family protein | F: TGCCACTCTACGTCTCTTCTT  R: GGTGATCCCTCTCTGCTCTATT | 101 |
| Medtr3g087510 | PLAT-plant-stress protein | F: GTCCTCATCATGGGTGGTATTG  R: GCAAGCCATTGTTCCACTTC | 100 |
| Medtr0121s0060 | Stress responsive A/B barrel  domain protein | F: CCCTCACATTCTCACCATTCA  R: CTTGGGAAGTAGAGGACATCTTG | 117 |
| Medtr2g028670 | Actin depolymerizing factor | F: GCATCTGGTATGGCAGTCC  R: GCACTCATCAGCAGGAAGG | 183 |
